# Meox1 Promotes Cardiac Fibrosis and Pathological Remodeling following Myocardial Infarction through Cthrc1/p-Smad2/3 Signaling

**DOI:** 10.7150/ijbs.113825

**Published:** 2026-01-01

**Authors:** Mian Zhang, Xiao-wen Meng, Yu-fan Yang, Xin-yu Chen, Yi-chan Wang, Jing-jie Wan, Jun Ding, Bi-ying Wang, Ke Peng, Fu-hai Ji

**Affiliations:** 1 Department of Anesthesiology, The First Affiliated Hospital of Soochow University, Suzhou 215006, China.; 2 Institute of Anesthesiology, Soochow University, Suzhou 215006, China.

**Keywords:** Meox1, Cthrc1, cardiac fibroblasts, cardiac fibrosis, myocardial infarction

## Abstract

**Aims:** Myocardial infarction (MI) induces pathological cardiac fibrosis and ventricular remodeling, which leads to cardiac dysfunction and heart failure. Mesenchyme homeobox 1 (Meox1) was shown to be an essential transcriptional switch in fibroblasts activation; however, whether Meox1 is involved in the fibrosis process following MI remains unknown. We aimed to explore the role of Meox1 in cardiac fibrosis and remodeling post-MI and its underlying mechanisms.

**Methods and results:** Herein, we identified that Meox1 was highly expressed in activated fibroblasts (myofibroblasts, Myofbs), in response to MI in mice or transforming growth factor beta 1 (TGF-β1) stimulation in primary cardiac fibroblasts (CFs). Knockdown of Meox1 in Myofbs remarkably attenuated cardiac fibrosis and adverse remodeling post-MI and improved cardiac function. *In vitro*, Meox1 silencing inhibited the activation, proliferation, migration and fibrotic gene expression of primary CFs, whereas Meox1 overexpression resulted in the opposite biological effects. Mechanistically, Meox1 transcriptionally activated collagen triple helix repeat containing 1 (Cthrc1), which further promoted downstream Smad2/3 phosphorylation, thereby leading to CFs-to-Myofbs conversion. Overexpression of Cthrc1 abolished the cardioprotective effects of Meox1 silencing in mice. Moreover, Cthrc1 knockdown in primary CFs suppressed the effects of Meox1 on facilitating the phosphorylation of Smad2/3 and profibrotic phenotypes.

**Conclusions:** Our study revealed the key regulatory role of Meox1 in promoting cardiac fibrosis and heart failure by inducing the transformation of CFs-to-Myofbs through activating Cthrc1/p-Smad2/3 post-MI. Therefore, Meox1/Cthrc1/p-Smad2/3 signaling pathway might be a promising therapeutic target for cardiac fibrosis and remodeling in MI patients.

## 1. Introduction

Ischemic heart disease remains the predominant cause of death globally, with 197 million prevalent cases and 9.14 million deaths in 2019, presenting a heavy economic and healthy burden 1-3. Due to the extremely limited regenerative capacity of adult mammalian hearts, dead cardiomyocytes caused by ischemia are replaced by fibrotic and remodeled tissues 4, 5. Fibrosis, characterized by the deposition of extracellular matrix (ECM) proteins, is a crucial physiological response involved in tissue repair after myocardial infarction (MI). During the post-infarction early stage, fibrosis is markedly induced in the infarct zone to form a stable scar, reinforcing the ventricles to prevent heart rupture. In the late phase of MI, however, prolonged activation of fibrotic pathways leads to excessive collagen-dominated ECM accumulation and scar formation, myocardial stiffness, cardiomyocyte hypertrophy, and reduced heart tissue compliance 6-8. This pathological fibrosis and remodeling disrupt cardiac electrical conduction, deteriorate contractile and diastolic function, and induce arrhythmias and hemodynamic abnormalities, ultimately leading to heart failure 9-11. Currently, there is no effective clinical therapy to mitigate the progression of post-MI fibrosis.

Cardiac fibroblasts (CFs), the most abundant stromal cells in the heart, remain quiescent and produce low levels of ECM proteins under normal conditions. Upon acute tissue injury or under chronic stress, CFs are activated and switch into myofibroblasts (Myofbs) to express abundant ECM proteins and secret various profibrotic factors 6, 12-14. Myofbs possess enhanced proliferative, migratory, and contractile capabilities and play a crucial role in cardiac fibrosis and adverse remodeling 7, 15, 16. Therefore, to limit post-MI excessive fibrotic response and improve cardiac remodeling, it is critical to have a better understanding of the specific mechanism of CFs activation following cardiac ischemia and identify novel targets to block the CFs-to-Myofbs phenotype transformation.

Mesenchyme homeobox 1 (Meox1), a member of the Hox gene subfamily, is widely expressed in mesodermal and mesenchymal-derived cells. Physiologically, Meox1 functions as a transcription factor to regulate cellular proliferation, differentiation and migration. Meox1 plays an essential role in the development of skeletal muscle and cardiovascular system, as well as in the formation of somite 17, 18. Previous studies showed that the increased expression of Meox1 was associated with various pathophysiological processes in human diseases, such as the invasion and metastasis of multiple tumors and the formation of hypertrophic skin scars 19-22. Recently, researchers have proposed that Meox1 may act as a transcriptional switch controlling fibroblast activation in cardiac diseases 23. Additionally, Meox1 has been reported to be associated with the expression of profibrotic genes (*Col1a1*, *Postn*, and *Tgfb1*) in the hearts of mice with chronic MI 24. However, whether Meox1 regulates post-MI CFs-to-Myofbs transformation and pathological remodeling and the specific mechanism are unknown.

In this study, we provided direct evidence demonstrating that downregulating Meox1 suppressed CFs-to-Myofbs transition, alleviated pathological fibrosis and remodeling, and improved cardiac function in mice post-MI, primarily through inhibiting the collagen triple helix repeat containing 1 (Cthrc1)/p-Smad2/3 signaling pathway. From the perspective of clinical translation, targeting Meox1/Cthrc1/p-Smad2/3 axis offers novel therapeutic potential for treating heart failure and improving the outcomes in patients with MI.

## 2. Methods

The detailed description of methods can be found in the **Supplementary Material**.

### 2.1 Animal

Healthy male C57BL/6J mice (8-10 weeks old) were purchased from Cavens Biogle Model Animal Research Co., Ltd. (Changzhou, China) and housed in a specific pathogen-free-grade facility with a controlled temperature and a 12 h light-dark cycle. All mice were allowed ad libitum access to food and water. The animal study protocol was reviewed and approved by the Animal Care and Use Committee of Soochow University (Ethic Certificate No: 202209A0021). Moreover, all perioperative care and surgical procedures were strictly performed in compliance with the National Institutes of Health Guideline Guide for the Care and Use of Laboratory Animals (NIH Publication No.85-23, revised 1996). At the end of the study, mice were euthanized by performing cervical dislocation under deep anesthesia with 3% pentobarbital sodium.

### 2.2 Western blot

Western blot analysis was performed as previously reported 11. More details regarding the procedure were described in the **Supplementary Material**. Detailed information about antibodies used for western blot was listed in the **Supplementary Material**, **Table S1**.

### 2.3 siRNA transfection

Specific small interfering RNAs (siRNAs) targeting Meox1 and negative control siRNA sequence were designed and synthesized by Sangon Biotech Co., Ltd. (Shanghai, China). Target sequence of Cthrc1 siRNAs and scramble control siRNA were designed and synthesized by RiboBio Co., Ltd. (Guangzhou, China). The detailed sequence information of siRNAs was shown in **Table S2**. Transient transfection of siRNA was performed using Lipofectamine® 2000 transfection reagent (Invitrogen, #11668019) following the manufacturer's instructions.

### 2.4 RT-qPCR

Total RNA was extracted from neonatal mice cardiac fibroblasts (NMCFs) using Trizol reagent according to the manufacturer's specifications. The primer sequences used in RT-qPCR were shown in **Table S3**. The **Supplementary Material** provides further details.

## 3. Results

### 3.1 MI-induced cardiac dysfunction and pathological remodeling in mice

We established the MI model in adult C57BL/6J mice and found that the survival rate was significantly reduced (60% vs. 93.3%) within 28 days in the MI group compared to the sham group (**Figure S1A**). Until 28 days following MI, the echocardiography results showed significant reductions in left ventricular ejection fraction (EF) and fractional shorting (FS) (**Figure S1B-S1D**; **Table S4**) and significant left ventricular dilation as reflected by the increased left ventricular internal diameter at end-systole (LVID,s) (**Figure S1E**; **Table S4**), without between-group difference in left ventricular internal diameter at end-diastole (LVID,d) (**Figure S1F**; **Table S4**).

The mice in the MI and sham groups had similar body weight during 28 days after MI (**Figure S1G**). The ratios of heart weight to body weight (HW/BW) and heart weight to tibia length (HW/TL) were substantially increased in the MI group over time (**Figure S1H** and** S1I**), demonstrating progressive cardiac hypertrophy caused by MI. At day 28, we observed notably increased scar circumference (**Figure S1J** and** S1K**) using the Masson staining, enlarged fibrotic area (**Figure S1L** and** S1M**) with the Picrosirius red staining, and greater cardiomyocyte cross-sectional area (**Figure S1N** and** S1O**) by the wheat germ agglutinin (WGA) staining. Additionally, immunohistochemical staining displayed extensive deposition of ECM proteins including Collagen I and Postn in cardiac tissue of MI mice (**Figure S1P**).

Western blot analysis of lysates from the left ventricular infarct zone showed that the protein expression of alpha-smooth muscle actin (α-SMA, a marker of Myofbs), transforming growth factor beta 1 (TGF-β1), Vimentin (a marker of fibroblast), and Collagen I were significantly upregulated on post-MI day 28, while the protein expression of matrix metalloproteinase 2 (MMP2), an enzyme known to degrade and remodel ECM, was significantly increased on days 7 and 14 and decreased to a normal level on day 28 (**Figure S1Q** and** S1R**). In the left ventricular remote zone, these fibrosis-related proteins did not show significant changes during 28 days after MI (except for increased expression of α-SMA and decreased Vimentin on day 7) (**Figure S2A-S2F**).

These results demonstrated that MI induced significant deterioration in cardiac function and adverse structural remodeling (cardiac hypertrophy, scar formation, and increased fibrosis). The progressive nature of these changes underscores the importance of early therapeutic interventions to mitigate post-MI cardiac dysfunction.

### 3.2 Upregulation of Meox1 in activated cardiac fibroblasts *in vivo* and *in vitro*

**Figure 1:**
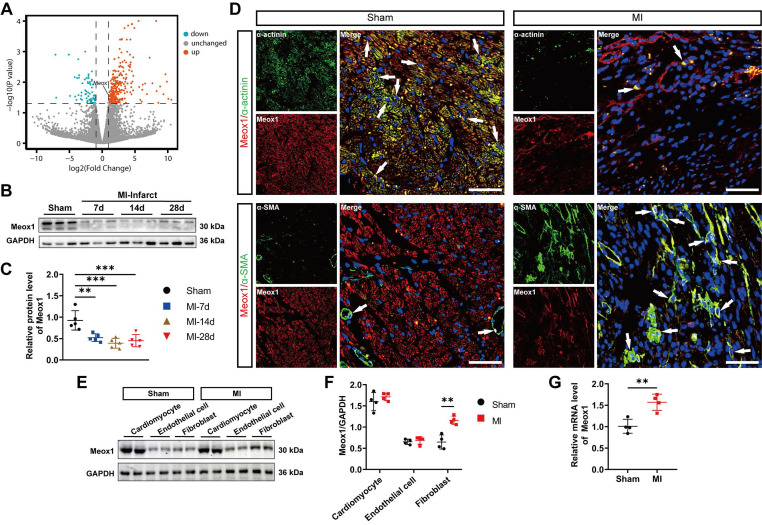
** Meox1 was induced in activated CFs in mouse hearts after MI. (A)** The volcano plot of DEGs in mouse CFs isolated from the uninjured and the infarcted left ventricle (GSE186079 dataset), in which Meox1 was upregulated.** (B, C)** Representative immunoblots and quantitative analysis of Meox1 protein level in infarcted heart tissues at indicated time points. n=5.** (D)** Representative immunofluorescence micrographs of co-localized expressions of Meox1 (red) and α-actinin (green)/α-SMA (green) in heart sections at day 28. Arrows indicate representative co-localizations. Scale bar, 50 μm. **(E, F)** Representative immunoblots and quantitative analyses of Meox1 protein levels in cardiomyocytes, endothelial cells and fibroblasts, respectively, isolated from hearts of adult mice subjected to sham or MI surgery for 28 days. n=4.** (G)** Quantitative analysis of Meox1 mRNA in isolated CFs at 28 days after MI or sham. n=4. Data are mean ± SD. One-way ANOVA followed by Dunnett post hoc test **(C)** and unpaired student's test **(F, G)**. **P<0.01, ***P<0.001.

To search for key factors that may regulate post-MI fibrosis and adverse ventricular remodeling, we analyzed differentially expressed genes (DEGs) in CFs isolated from left ventricle of the sham and MI mice using a publicly available dataset, GSE186079 (**Table S5**). A total of 345 DEGs were identified (268 upregulated and 77 downregulated) (**Figure 1A**). Among the significantly upregulated genes, we shortlisted transcriptional factor-encoding genes, yielding 11 candidates (*Meox1, Erg, Runx1, Nfatc1, Rarg, Cc2d1a, Vdr, Atoh8, Atf5, Id1, Hif1a*) that may mediate robust transcriptional responses. Given that the prior seminal work by Alexanian et al. revealing the critical role of Meox1 in pressure overload-induced cardiac fibrosis 23, we wondered whether it also regulated the activation of fibroblasts in post-MI remodeling and fibrosis. Thus, we first examined the expression of Meox1 in our model using western blot, showing that its protein level was remarkably reduced in infarct tissue during 28 days after MI (**Figure 1B** and** 1C**), but not in the left ventricular remote zone (**Figure S2G**). Then the immunofluorescence staining results showed that the Meox1/α-actinin co-localization was reduced, while the Meox1/α-SMA co-localization was increased in cardiac tissue after MI (**Figure 1D**). Given the loss of cardiomyocytes following MI, we proceeded to isolate cardiomyocytes, endothelial cells and CFs from the hearts of adult mice to assess the differential expression of Meox1 across these cardiac cell populations. Our findings demonstrated that Meox1 expression was notably higher in cardiomyocytes compared to endothelial cells and CFs. Furthermore, we observed a significant upregulation of Meox1 expression in CFs isolated from MI mice compared to sham mice. In contrast, Meox1 expression levels remained unchanged in both cardiomyocytes and endothelial cells across the two groups (**Figure 1E** and **1F**). Additionally, RT-qPCR analysis confirmed the increased expression of Meox1 mRNA in CFs isolated from MI mice (**Figure 1G**). Hence, these evidences suggested that Meox1 was predominantly expressed in cardiomyocytes under normal conditions and upregulated in activated CFs after MI.

Next, we further isolated primary fibroblasts from hearts of neonatal mice, and a high purity of NMCFs was confirmed by positive staining with Vimentin and negative expression of CD31 (a marker of endothelial cells) (**Figure S3A**). TGF-β1, as the most effective mediator for quiescent fibroblasts transformation into activated fibroblasts, has been widely used to mimic the fundamental biological process leading to fibrotic diseases *in vitro* 25, 26. We observed that the expression of fibrosis-related genes, including α-SMA, Vimentin, TGF-β1, Fibronectin-1, Collagen I, Postn and lysyl oxidase (LOX), were all significantly elevated in NMCFs after TGF-β1 treatment (**Figure S3B-S3D**). Importantly, TGF-β1 stimulation significantly upregulated the protein and mRNA expression of Meox1 in NMCFs (**Figure S3B-S3D**). Moreover, the immunofluorescence results showed that the increased Meox1 was mainly located in nuclei (**Figure S3E** and** S3F**). Additionally, the NMCFs showed enhanced proliferation (**Figure S3G**) and migration (**Figure S3H** and** S3I**) after TGF-β1 treatment. These data indicated the successful establishment of the *in vitro* fibrotic model and the upregulation of Meox1 in NMCFs following TGF-β1 stimulation.

### 3.3 Meox1-mediated NMCFs-to-Myofbs conversion *in vitro*

**Figure 2:**
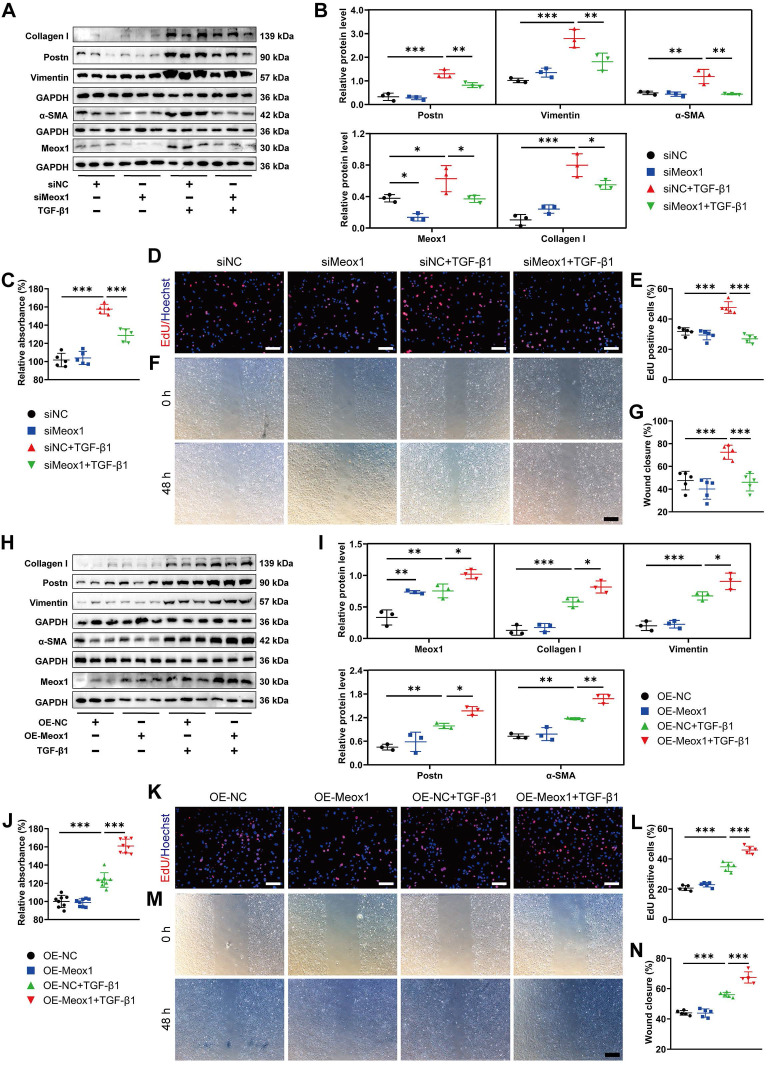
** Meox1 modulated CFs-to-Myofbs phenotype transformation upon TGF-β1 stimulation. (A, B)** Representative immunoblot images and quantitative analyses of Postn, Vimentin, α-SMA, Meox1 and Collagen I protein levels in NMCFs transfected with siMeox1 or siNC followed by exposure to vehicle or TGF-β1. n=3. **(C)** Proliferation of NMCFs determined by CCK-8 assay. n=5. **(D, E)** EdU assay and quantitative analysis of the percentage of EdU-positive cells (red). Scale bar, 100 μm. n=5. **(F, G)** Representative scratch micrographs of NMCFs transfected with siMeox1 or siNC at 0 and 48 h after vehicle or TGF-β1 treatment and quantification of the area of scratch wound closure. Scale bar, 500 μm. n=5. **(H, I)** Representative immunoblots and quantitative analyses of Meox1, Collagen I, Vimentin, Postn and α-SMA protein levels in NMCFs transfected with OE-Meox1 or OE-NC followed by exposure to vehicle or TGF-β1. n=3. **(J)** Proliferation of NMCFs determined by CCK-8 assay. n=8. **(K, L)** EdU assay and quantitative analysis of the percentage of EdU-positive cells (red). Scale bar, 100 μm. n=5. **(M, N)** Representative scratch micrographs of NMCFs transfected with OE-Meox1 or OE-NC at 0 and 48 h after vehicle or TGF-β1 treatment and quantification of the area of scratch wound closure. Scale bar, 500 μm. n=5. Data are mean ± SD. *P<0.05, **P<0.01, ***P<0.001 by one-way ANOVA followed by Dunnett post hoc test.

To further investigate whether Meox1 was involved in TGF-β1-induced CFs activation, we manipulated the expression of Meox1 in NMCFs through transfection with small interfering RNA (siRNA) and plasmid specific for Meox1. Significantly decreased Meox1 mRNA and protein expression levels were validated in NMCFs transfected with siMeox1 (**Figure S4A-S4C**). Western blot analysis showed that knockdown of Meox1 did not change the expression of fibrotic proteins (Postn, Vimentin, α-SMA and Collagen I) in NMCFs under basal condition; however, the TGF-β1-induced upregulation of these proteins was significantly reversed by siMeox1 (**Figure 2A** and** 2B**). Cell counting kit-8 (CCK-8) assay and 5-ethynyl-2'-deoxyuridine (EdU) assay showed that cellular proliferation induced by TGF-β1 was significantly suppressed by siMeox1 (**Figure 2C-2E**). Additionally, Meox1 silencing remarkably inhibited the migration of NMCFs in response to TGF-β1 in the scratch healing assay (**Figure 2F** and** 2G**). Conversely, we found that Meox1 overexpression further increased the protein levels of Postn, Vimentin, α-SMA and Collagen I (**Figure 2H** and** 2I**) and enhanced proliferation (**Figure 2J-2L**) and migration (**Figure 2M** and** 2N**) in NMCFs exposed to TGF-β1. Taken together, these results suggested that Meox1 participated in the regulation of TGF-β1-induced NMCFs-to-Myofbs conversion.

### 3.4 Attenuation of post-MI cardiac dysfunction and pathological remodeling by Meox1 knockdown in Myofbs

**Figure 3:**
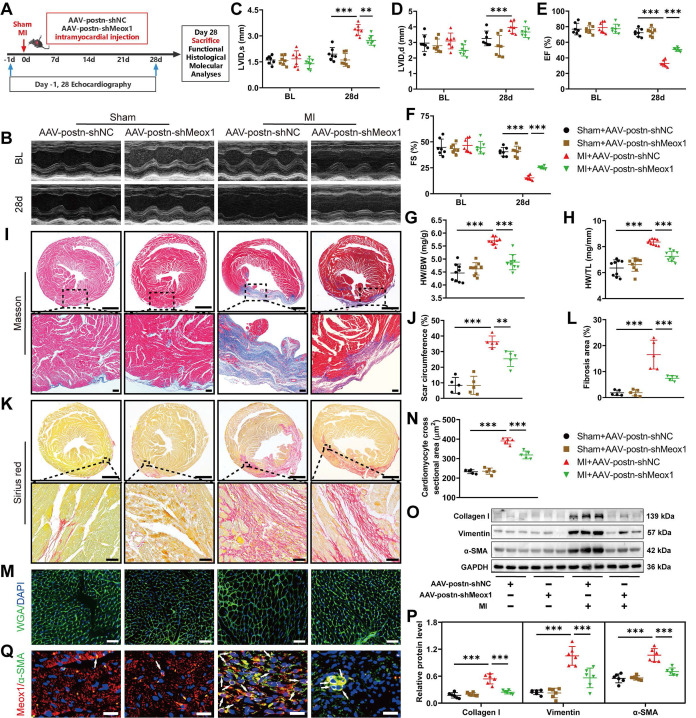
** Myofbs-specific Meox1 knockdown ameliorated post-MI cardiac dysfunction, adverse remodeling, and cardiac fibrosis. (A)** Schematic diagram of AAV-postn-shMeox1 and AAV-postn-shNC. **(B)** Representative images of M-mode echocardiography at baseline and on day 28. **(C-F)** LVID,s, LVID,d, LVEF, and LVFS. n=7. **(G, H)** The ratios of HW to BW and HW to TL on day 28. n=9. **(I, J)** Representative images and quantification of scar circumference of heart sections. Scale bars, 1000 μm (upper) and 100 μm (lower). n=5. **(K, L)** Representative images and quantification of fibrotic area of heart sections. Scale bars, 1000 μm (upper) and 50 μm (lower). n=5. **(M, N)** Representative images and quantification of cross-sectional area of cardiomyocyte in heart sections. Scale bar, 50 μm. n=5. **(O, P)** Representative immunoblots and quantitative analyses of Collagen I, Vimentin and α-SMA protein levels in heart tissues. n=6. **(Q)** Representative immunofluorescence micrographs of Meox1 (red), α-SMA (green) and DAPI (blue) in heart tissues. Arrows indicate representative co-localizations of Meox1 and α-SMA. Scale bar, 20 μm. Data are mean ± SD. *P<0.05, **P<0.01, ***P<0.001 by one-way ANOVA followed by Dunnett post hoc test.

Postn, an ECM protein, is strongly produced in Myofbs rather than other cell types in injured hearts 27, 28. Postn promoter has been routinely used to specifically modulate gene expression in Myofbs 29, 30. To explore the effect of Meox1 in Myofbs on post-MI cardiac dysfunction, fibrosis and pathological remodeling, we generated adeno-associated virus 9 (AAV9) particles carrying the negative control shRNA or Meox1 shRNA under the control of postn promoter (AAV-postn-shNC or AAV-postn-shMeox1). Theses AAV particles were delivered into the left ventricular wall via intramyocardial injection at 4-to-5 separate points (**Figure 3A**).

To detect the efficacy of knockdown of Meox1 in CFs, we isolated the cells from the heart of adult mice subjected to MI surgery for 28 days following injection of AAV. The results showed that Meox1 was significantly knockdown at both mRNA and protein levels in isolated CFs (**Figure S5A-S5C**). Then, we examined the role of Meox1 on MI model mice. Before the sham and MI procedures, all mouse hearts in four groups had similar ventricular dimensions and contractile function as determined by echocardiography (**Figure 3B-3F** and **Table S6**). Compared with the sham+AAV-postn-shNC group, the MI+AAV-postn-shNC group exhibited significantly increased LVID,s (**Figure 3C**) and decreased EF (**Figure 3E**) and FS (**Figure 3F**) at day 28, whereas these changes were blocked in the MI+AAV-postn-shMeox1. However, the increased LVID,d in MI mice was not affected by AAV-postn-shMeox1 (**Figure 3D**).

The increased ratios of HW/BW and HW/TL after MI were significantly reduced in the MI+AAV-postn-shMeox1 group (**Figure 3G** and** 3H**). Moreover, we observed notably decreased scar circumference (**Figure 3I** and** 3J**), shrunken interstitial fibrotic area (**Figure 3K** and** 3L**), and smaller cardiomyocyte cross-sectional area (**Figure 3M** and** 3N**) in the MI+AAV-postn-shMeox1 group compared to the MI+AAV-postn-shNC group. Consistent with the results of histological staining, western blot analysis confirmed that the upregulated protein levels of Collagen I, Vimentin and α-SMA in post-MI tissues were remarkedly decreased in the MI+AAV-postn-shMeox1 group (**Figure 3O** and** 3P**). Additionally, immunofluorescence staining presented decreased co-localization of α-SMA and Meox1 in the infarct hearts with AAV-postn-shMeox1 (**Figure 3Q**).

These *in vivo* results indicated that Myofbs-specific deletion of Meox1 prevented the deterioration of cardiac function and attenuated ventricular fibrosis and adverse remodeling in mice following MI.

### 3.5 Meox1 regulating Cthrc1 expression underlying pathological fibrosis

**Figure 4:**
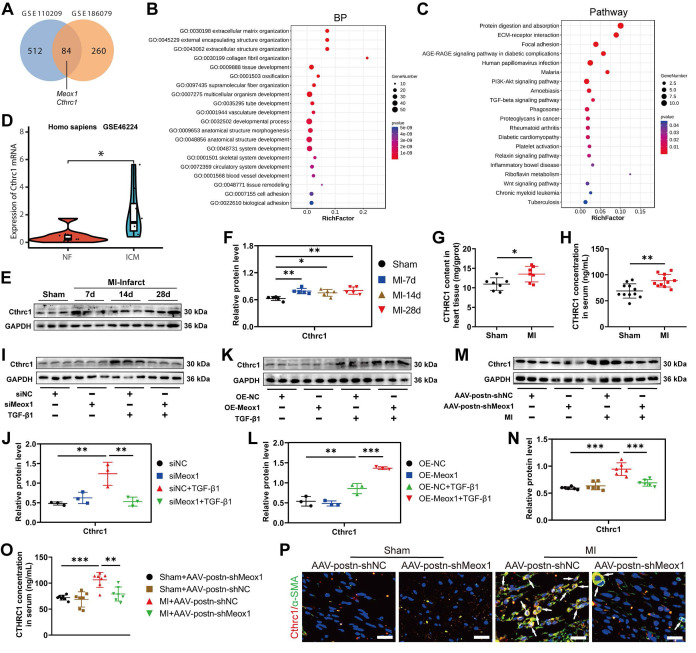
** Meox1 regulated the expression of Cthrc1 in response to pathological fibrosis stimulation. (A)** Venn diagram showing the overlap of DEGs between GSE110209 and GSE186079 datasets. **(B)** Top 20 biological process categories in the GO study of the common DEGs. **(C)** Top 20 pathways in the KEGG pathway enrichment analysis of the common DEGs. **(D)** The expression of Cthrc1 in the left ventricular tissues from non-failing (NF) and ischemic cardiomyopathy (ICM) hearts (GSE46224 dataset). **(E-F)** Representative immunoblots and quantitative analysis of Cthrc1 protein level in infarcted mouse heart tissues. n=5. **(G, H)** Cthrc1 concentrations in mouse heart tissue (n=7) and serum (n=11) at day 28 detected by ELISA. **(I, J)** Representative immunoblots and quantitative analysis of Cthrc1 protein level in NMCFs transfected with siMeox1 or siNC followed by exposure to vehicle or TGF-β1. n=3. **(K, L)** Representative immunoblots and quantitative analysis of Cthrc1 protein level in NMCFs transfected with OE-Meox1 or OE-NC followed by exposure to vehicle or TGF-β1. **(M, N)** Representative immunoblots and quantitative analysis of Cthrc1 protein level in mouse heart tissues at day 28. n=6. **(O)** Cthrc1 concentration in mouse serum at day 28. n=7. **(P)** Representative micrographs of immunofluorescence staining with Cthrc1 (red), α-SMA (green) and DAPI (blue) in mouse heart tissues at day 28. Arrows indicate representative co-localizations of Cthrc1 and α-SMA. Scale bar, 20 μm. n=3. Data are mean ± SD. One-way ANOVA followed by Dunnett post hoc test **(F, J, L, N, O)** and unpaired student's test **(G, H)**. *P<0.05, **P<0.01, ***P<0.001.

To explore the potential downstream target gene of Meox1 on regulating CFs activation and cardiac fibrosis, we performed transcriptomic analysis using the data obtained from GSE110209 and GSE186079 (**Table S5**). In ventricular tissues between the sham and MI mice, we identified 84 common DEGs (**Figure 4A**; **Table S7**), among which Meox1 and collagen triple helix repeat containing 1 (Cthrc1) were upregulated in both post-MI tissue (**Figure S6A**) and isolated CFs (**Figure S6B**). Cthrc1 is a secreted protein that has been proved to affect the deposition and synthesis of ECM molecules and promote the proliferation of CFs 28, 31.

Gene ontology (GO) analysis of the common DEGs revealed that the mainly enriched biological processes (**Figure 4B**), molecular function (**Figure S6C**) and cellular component (**Figure S6D**) included ECM organization, ECM structural constituent, collagen-containing ECM, collagen fiber organization, etc., all of which are known to be closely associated with cardiac remodeling. In addition, Kyoto Encyclopedia of Genes and Genomes (KEGG) pathway analysis revealed significant enrichment in the pathways of protein digestion and absorption, ECM-receptor interaction, and focal adhesion (**Figure 4C**).

Moreover, we analyzed data from GSE202228 (**Table S5**) and found that Cthrc1 was significantly upregulated in left ventricular tissues of MI rats, accompanied by upregulated expression of Meox1 (**Figure S6E**). To further confirm the increased Cthrc1 expression in ischemic heart disease, we analyzed patients' data from GSE46224 dataset (**Table S5**). The results showed that Cthrc1 was obviously upregulated in the hearts of patients with ischemic cardiomyopathy compared to non-failing hearts (**Figure 4D**).

Consistent with the sequencing data, we observed a significant increase in Cthrc1 protein expression in infarct tissues of the MI mice on days 7, 14 and 28 (**Figure 4E** and** 4F**), while this change was not found in the left ventricular remote tissues (**Figure S7A** and** S7B**). The enzyme-linked immunosorbent assay (ELISA) results also showed that the Cthrc1 level was increased in MI mice, in both heart tissues (**Figure 4G**) and serum (**Figure 4H**). Additionally, immunofluorescence staining showed that the increased Cthrc1 was predominantly co-localized with α-SMA in infarct hearts (**Figure S7C**).

Subsequently, we applied Meox1 silencing and overexpression in NMCFs under TGF-β1 stimulation. We found that siMeox1 evidently restrained the increased expression of Cthrc1 protein induced by TGF-β1 by western blot (**Figure 4I** and** 4J**) and immunofluorescence staining (**Figure S8A**). Conversely, overexpression of Meox1 resulted in further increased Cthrc1 protein expression (**Figure 4K** and** 4L**) and enhanced fluorescent intensity of α-SMA and Cthrc1 (**Figure S8B**). In MI mice, knockdown of Meox1 using AAV-postn-shMeox1 led to a significant reduction in Cthrc1 protein level by western blot (**Figure 4M** and** 4N**) and ELISA (**Figure 4O**). Immunofluorescence analysis revealed decreased co-localization of Cthrc1 and α-SMA in the infarct zone of MI+AAV-postn-shMeox1 mice compared with MI+AAV-postn-shNC mice (**Figure 4P**).

Collectively, these findings suggested that Cthrc1 expression may be a conserved molecular signature of post-MI fibrosis and remodeling. These *in vivo* and *in vitro* results provided direct evidence that Meox1 facilitated the expression of Cthrc1 in Myofbs in response to pathological fibrosis stimulation.

### 3.6 Suppression of CFs-to-Myofbs conversion by Cthrc1 silencing *in vitro*

To examine the role of Cthrc1 in CFs differentiation, we specifically inhibited Cthrc1 expression in NMCFs by siRNA. After siCthrc1 transfection of NMCFs, both the mRNA and protein expression of Cthrc1 was effectively blocked (**Figure S9A-S9C**). The results of western blot showed that siCthrc1 had no impact on the expression of fibrotic genes (Collagen I, Postn, Vimentin and α-SMA) in the absence of external TGF-β1 stimulation; however, the increased expression of these fibrotic proteins under TGF-β1 exposure was blocked by siCthrc1 (**Figure S10A** and** S10B**). Similarly, immunofluorescence assay showed that Cthrc1 knockdown suppressed the fluorescent intensity of TGF-β1-induced α-SMA in NMCFs (**Figure S10C**). Moreover, the enhanced proliferation and migration of NMCFs induced by TGF-β1 were significantly restricted by siCthrc1, as confirmed by CCK-8 (**Figure S10D**), EdU (**Figure S10E** and** S10F**) and scratch healing assay (**Figure S10G** and** S10H**). These data indicated that Cthrc1 played a key role in the regulation of CFs-to-Myofbs conversion upon TGF-β1 stimulation.

**Figure 5:**
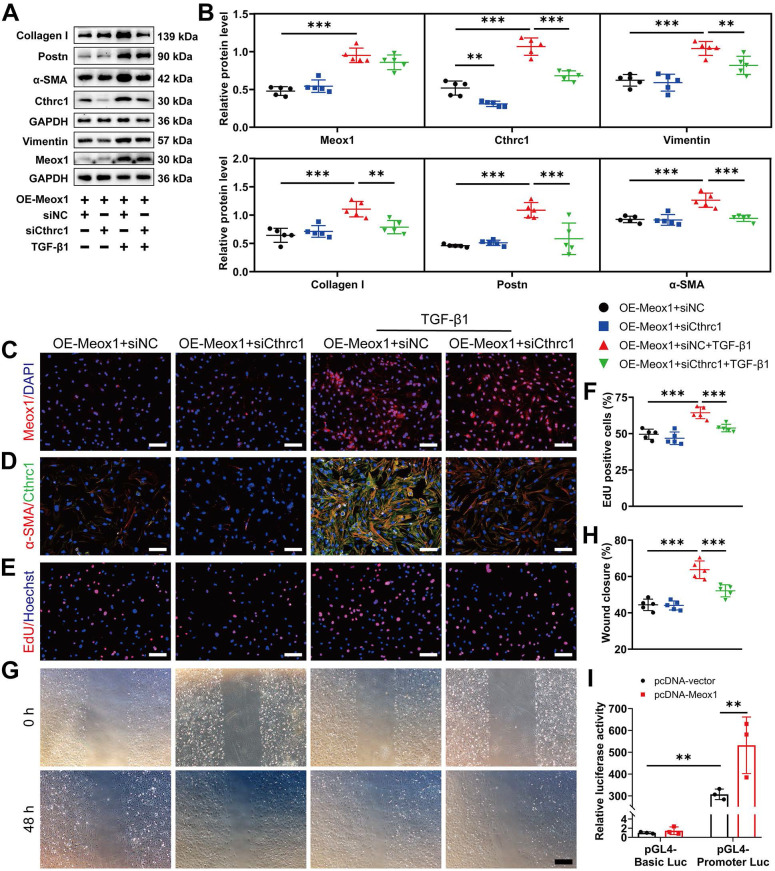
** Silencing of Cthrc1 reversed the stimulative effect of Meox1 on CFs-to-Myofbs phenotype transformation *in vitro*. (A, B)** Representative immunoblots and quantitative analyses of Meox1, Cthrc1, Collagen I, Postn, Vimentin and α-SMA protein levels in NMCFs co-transfected with OE-Meox1 and siCthrc1 or siNC after exposure to vehicle or TGF-β1. n=5. **(C, D)** Representative images of immunofluorescent staining of Meox1 (red)**/**α-SMA (red) and Cthrc1 (green) in NMCFs. Scale bar, 100 μm. **(E)** Representative micrographs of EdU staining and quantitative analysis of the percentage of EdU-positive cells (red). Scale bar, 100 μm. n=5. **(G, H)** Representative micrographs of scratch wound of NMCFs at 0 and 48 h after vehicle or TGF-β1 treatment and quantification of the area of scratch wound closure. Scale bar, 500 μm. n=5. **(I)** Dual luciferase activities in HEK293T cells co-transfected with pGL4-luciferase reporter vectors containing Cthrc1 promoter or not and Meox1 plasmid or control plasmid. n=3. Data are mean ± SD. **P<0.01, ***P<0.001 by one-way ANOVA followed by Dunnett post hoc test.

To further determine the involvement of Cthrc1 in Meox1-regulated Myofbs phenotypes, we inhibited Cthrc1 expression in NMCFs overexpressing Meox1. Western blot analysis showed that the protein expression of Cthrc1, Vimentin, Collagen I, Postn, and α-SMA was significantly decreased in the OE-Meox1+siCthrc1+TGF-β1 group, compared to the OE-Meox1+siNC+TGF-β1 group (**Figure 5A** and** 5B**). However, in Meox1-overexpressed NMCFs upon TGF-β1 stimulation, Cthrc1 silencing did not alter Meox1 protein expression (**Figure 5A** and** 5B**) and nuclear translocation (**Figure 5C**). Immunofluorescence staining revealed that the enhanced fluorescent intensity of α-SMA and Cthrc1 under Meox1 overexpression were decreased after siCthrc1 (**Figure 5D**). Cthrc1 silencing significantly abolished the enhanced proliferation (**Figure 5E** and** 5F**) and migration (**Figure 5G** and** 5H**) of NMCFs overexpressing Meox1. Furthermore, dual-luciferase reporter assay showed that Meox1 overexpression significantly enhanced the luciferase activity of Cthrc1 promoter (**Figure 5I**), suggesting a positive regulation of Cthrc1 transcription by Meox1. Collectively, these data suggested that Meox1 promoted TGF-β1-induced CFs-to-Myofbs transformation via regulating Cthrc1 expression transcriptionally.

### 3.7 Cthrc1 overexpression reverses cardioprotective and antifibrotic effects of Meox1 knockdown after MI

**Figure 6:**
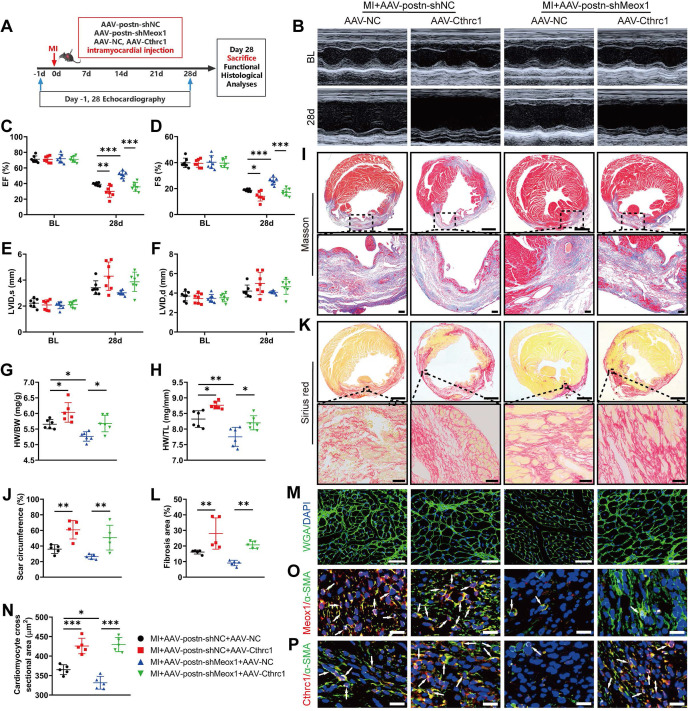
** Cthrc1 overexpression blocked cardioprotective and antifibrotic effects of Meox1 knockdown after MI. (A)** Schematic diagram of AAV-Cthrc1 or AAV-NC and AAV-postn-shMeox1 or AAV-postn-shNC after MI. **(B)** Representative images of M-mode echocardiography at baseline and 28 days after MI. **(C-F)** LVEF, LVFS, LVID,s, and LVID,d. n=7. **(G, H)** The ratios of HW to BW and HW to TL. n=6. **(I, J)** Representative images and quantification of scar circumference of heart sections. Scale bars, 1000 μm (upper) and 100 μm (lower). n=5. **(K, L)** Representative images and quantification of fibrotic area of heart sections. Scale bars, 1000 μm (upper) and 50 μm (lower). n=5. **(M, N)** Representative images and quantification of cross-sectional area of cardiomyocyte of heart sections. Scale bar, 50 μm. n=5. **(O, P)** Representative immunofluorescence micrographs of co-localized expressions of Meox1 (red)/Cthrc1 (red) and α-SMA (green). Arrows indicate representative co-localizations. Scale bar, 20 μm. Data are mean ± SD. *P<0.05, **P<0.01, ***P<0.001 by one-way ANOVA followed by Dunnett post hoc test.

To identify whether the effect of Meox1 knockdown on ameliorating cardiac dysfunction and adverse remodeling was primarily mediated through inhibiting Cthrc1 expression, we delivered Cthrc1 expressing AAV (AAV-Cthrc1) and/or AAV-postn-shMeox1 particles into the peri-infarct zone of mouse hearts (**Figure 6A**).

Prior to model establishment, the overexpression efficiency of Cthrc1 was confirmed using western blot (**Figure S11A** and **S11B**). Compared with the MI+AAV-postn-shNC+AAV-NC group, the echocardiographic results showed significantly lower EF and FS in the MI+AAV-postn-shNC+AAV-Cthrc1 group at day 28 (**Figure 6B-6D**; **Table S8**), without between-group differences in ventricular dilation parameters (LVID,s and LVID,d) (**Figure 6E** and** 6F**; **Table S8**). Moreover, the ratios of HW/BW and HW/TL were significantly elevated in the MI+AAV-postn-shNC+AAV-Cthrc1 group (**Figure 6G** and** 6H**). In addition, histological staining showed that Cthrc1 overexpression markedly increased scar circumference (**Figure 6I** and** 6J**), fibrotic area (**Figure 6K** and** 6L**) and cardiomyocyte cross-sectional area (**Figure 6M** and** 6N**). Immunofluorescence staining revealed that the co-localized expression of Meox1 and α-SMA was unchanged (**Figure 6O**) and the co-localized expression of Cthrc1 and α-SMA was increased (**Figure 6P**) in the MI+AAV-postn-shNC+AAV-Cthrc1 group. Therefore, these results showed that overexpression of Cthrc1 further exacerbated cardiac dysfunction, fibrosis, and pathological remodeling.

More importantly, Meox1 knockdown in the MI+AAV-postn-shMeox1+AAV-NC group increased the values of EF and FS (**Figure 6C** and** 6D**) and inhibited the ratios of HW/BW and HW/TL (**Figure 6G** and** 6H**) compared to the MI+AAV-postn-shNC+AAV-NC group. However, these effects were reversed by Cthrc1 overexpression in the MI+AAV-postn-shMeox1+AAV-Cthrc1 group. Cthrc1 overexpression also significantly increased scar circumference (**Figure 6I** and** 6J**), fibrotic area (**Figure 6K** and** 6L**), and cardiomyocyte cross-sectional area (**Figure 6M** and** 6N**) in the presence of Myofbs-specific Meox1 knockdown. Immunofluorescence staining showed that the co-localized expression of Cthrc1 and α-SMA was increased in the MI+AAV-postn-shMeox1+AAV-Cthrc1 group (**Figure 6P**).

All together, these *in vivo* results illustrated that knockdown of Meox1 exerted cardioprotective and anti-fibrotic effects following MI through suppressing the expression of Cthrc1.

### 3.8 Cthrc1 knockdown blocked the aggravation of cardiac dysfunction and cardiac fibrosis in Meox1-overexpressing mice

To further validate whether Meox1 promoted CFs activation and exerted profibrotic effects by increasing Cthrc1 expression *in vivo*, we injected Meox1 expressing AAV with the control of postn promoter (AAV-postn-Meox1) and/or AAV carrying Cthrc1 shRNA (AAV-shCthrc1) particles in mouse hearts immediately following MI.

The overexpression efficiency of Meox1 in CFs isolated from MI mice was confirmed using western blot (**Figure S11C** and** S11D**). A notable reduction of Cthrc1 expression in cardiac tissue was observed at day 28 following AAV-shCthrc1 transfection (**Figure S11E** and** S11F**). The echocardiographic results showed that Meox1 overexpression in CFs could further deteriorate cardiac systolic function, as evidenced by decreased EF and FS and increased LVID,s at day 28 (**Figure S12A**-**S12E**; **Table S9**). However, no difference was found in LVID,d between the MI+AAV-postn-NC+AAV-shNC group and the MI+AAV-postn-Meox1+AAV-shNC group (**Figure S12F**; **Table S9**). The ratios of HW/BW and HW/TL were significantly elevated in the MI+AAV-postn-Meox1+AAV-shNC group, compared with the MI+AAV-postn-NC+AAV-shNC group (**Figure S12G** and** S12H**). Moreover, histological staining showed that Meox1 overexpression markedly increased scar circumference (**Figure S12I** and** S12J**), fibrotic area (**Figure S12K** and** S12L**) and cardiomyocyte cross-sectional area (**Figure S12M** and** S12N**). These findings indicated that overexpression of Meox1 in CFs could promote progressive cardiac dysfunction and widespread cardiac fibrosis and remodeling after MI.

Oppositely, the MI+AAV-postn-NC+AAV-shCthrc1 group had significantly higher EF and FS (**Figure S12C** and** S12D**; **Table S9**), lower ratios of HW/BW and HW/TL (**Figure S12G** and** S12H**), and smaller scar circumference (**Figure S12I** and** S12J**), fibrotic area (**Figure S12K** and** S12L**) and cardiomyocyte cross-sectional area (**Figure S12M** and** S12N**), compared with the MI+AAV-postn-NC+AAV-shNC group. Thus, these *in vivo* data demonstrated that knockdown of Cthrc1 preserved cardiac function and prevented cardiac fibrosis and adverse remodeling in mice following MI.

Notably, we discovered significant increases in EF and FS, and decreases in LVID,s, HW/BW ratio, HW/TL ratio, scar circumference, fibrotic area and cardiomyocyte cross-sectional area in the MI+AAV-postn-Meox1+AAV-shCthrc1 group when comparing to the MI+AAV-postn-Meox1+AAV-shNC group (**Figure S12C**-**S12N**; **Table S9**). Taken together, it could be concluded that Cthrc1 knockdown protected against deteriorating cardiac dysfunction and pathological cardiac fibrosis and remodeling following MI in Meox1-overexpressing mice, pointing towards a potential therapeutic strategy to alleviate the adverse effects of Meox1 overexpression on cardiac function.

### 3.9 Meox1 promotes CFs activation by facilitating phosphorylation of Smad2/3 through regulating Cthrc1* in vivo* and *in vitro*

Phosphorylated Smad2/3, an important intracellular signaling in fibroblasts activation, has been reported to be persistently upregulated in infarct zone after myocardial ischemia and contribute to progressive cardiac fibrosis and functional deterioration 13, 32. Moreover, previous studies proposed that Cthrc1 may affect the phosphorylation level of Smad2/3 33-36. Therefore, we examined the effect of Cthrc1 on Smad2/3 phosphorylation in TGF-β1-treated NMCFs and mouse infarct hearts to investigate whether p-Smad2/3 is a potential downstream effector in Cthrc1-regulated CFs-to-Myofbs conversion.

**Figure 7:**
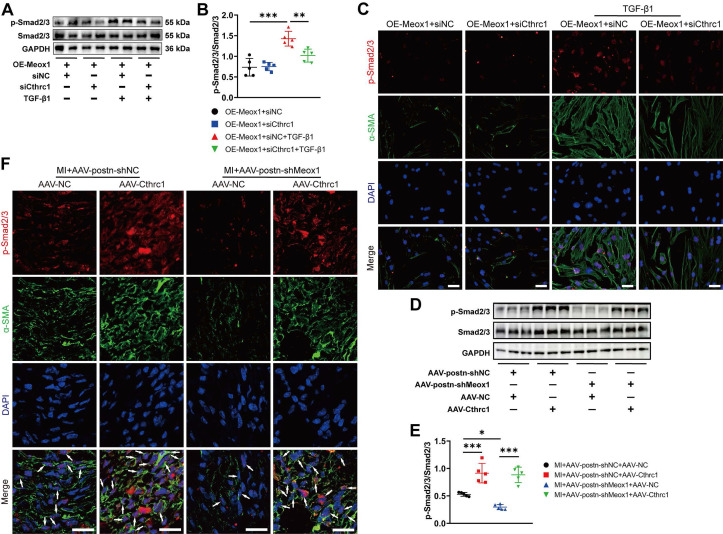
** Meox1 promoted CFs-to-Myofbs conversion by facilitating phosphorylation of Smad2/3 through regulating Cthrc1* in vitro* and* in vivo*. (A, B)** Representative immunoblots and quantitative analysis of p-Smad2/3 protein level in NMCFs co-transfected with OE-Meox1 and siCthrc1 or siNC after exposure to vehicle or TGF-β1. n=5. **(C)** Representative images of immunofluorescence double-staining of p-Smad2/3 (red) and α-SMA (green) in NMCFs. Scale bar, 50 μm. **(D, E)** Representative immunoblots and quantitative analysis of p-Smad2/3 protein level in the mouse heart tissues on day 28 following MI in the AAV-Cthrc1 or AAV-NC and AAV-postn-shMeox1 or AAV-postn-shNC groups. n=5. **(F)** Representative immunofluorescence micrographs of co-localized expression of p-Smad2/3 (red) and α-SMA (green) in the mouse heart sections on day 28 following MI. Arrows indicate representative co-localizations. Scale bar, 20 μm. Data are mean ± SD. *P<0.05, **P<0.01, ***P<0.001 by one-way ANOVA followed by Dunnett post hoc test.

Western blot assay showed that Cthrc1 silencing significantly blocked TGF-β1-induced p-Smad2/3 in NMCFs overexpressing Meox1 (**Figure 7A** and** 7B**). Subsequently, immunofluorescence staining revealed that Cthrc1 silencing in the OE-Meox1+siCthrc1+TGF-β1 group decreased the nuclear translocation of p-Smad2/3 and fluorescent intensity of α-SMA compared to the OE-Meox1+siNC+TGF-β1 group (**Figure 7C**). Additionally, we observed that the protein level of p-Smad2/3 (**Figure 7D** and** 7E**), and the co-localized expression of p-Smad2/3 and α-SMA (**Figure 7F**) were both decreased in the MI+AAV-postn-shMeox1+AAV-NC group compared with the MI+AAV-postn-shNC+AAV-NC group, which were abolished by the cotreatment with Cthrc1 overexpression in the MI+AAV-postn-shMeox1+AAV-Cthrc1 group (**Figure 7D-7F**).

Consequently, these data indicated that Meox1 promoted the activation of CFs via facilitating Cthrc1/p-Smad2/3 signaling.

## 4. Discussion

The abnormal fibrosis formation in hearts usually leads to pathological cardiac remodeling and heart failure. Therefore, the identification of underlying molecular targets of this process is of great importance for improving prophylactic and therapeutic strategies. In the present study, we found that Meox1 was a novel factor promoting post-MI adverse cardiac fibrosis and ventricular remodeling, and the upregulated Meox1 was exclusively expressed in CFs. The experimental results *in vivo* and *in vitro* showed that Meox1 knockdown blocked the activation of CFs and ameliorated cardiac fibrosis and dysfunction induced by MI. Mechanistically, the effects of Meox1 on CFs activation and fibrosis were mediated through Cthrc1 and its downstream Smad2/3 signaling. Specifically, the upregulated Meox1 transcriptionally activated Cthrc1 expression, which then promoted Smad2/3 phosphorylation, thus enhancing the proliferation, migration and synthesis of ECM proteins in Myofbs to accelerate the process of cardiac fibrosis. Taken together, our results demonstrated that inhibiting Meox1/Cthrc1/p-Smad2/3 signaling suppressed CFs-to-Myofbs conversion and prevented fibrotic remodeling and heart failure, which may provide clinical translational implications for improving the prognosis of MI patients.

Meox1, a highly conserved member of the Hox gene subfamily in mammals, encodes the Meox1 protein that recognizes and binds to specific DNA motifs in promoters of target genes, thereby regulating the transcription of these genes 17, 37. Previous studies have shown that Meox1 controls embryonic development through strictly modulating cellular proliferation and organogenesis 38, 39. During the remodeling process following vascular injury, the increased Meox1 regulated multiple signaling pathways to promote phenotypic transition in smooth muscle cells 18, 40. In addition, Meox1 also promoted the invasion, proliferation and metastasis of tumor cells, eventually accelerating the malignant progress of various cancers 19-21. Given the vital role of Meox1 in controlling cell fate and disease progression, it is necessary to systematically explore the role and mechanism of Meox1 in chronic heart diseases.

It has been reported that Meox1 was significantly increased in pathological cardiac remodeling upon pressure overload 41, and the overexpression of Meox1 in cardiomyocytes directly impaired cardiac function and accelerated cardiac hypertrophy and heart failure 37. Deepak et al. found that Meox1 was a central regulator of fibroblast activation associated with cardiac dysfunction after transverse aortic constriction 23. Moreover, a single-cell RNA sequencing (scRNA-seq) in human hearts revealed an abnormal increase in transcription factor Meox1 in CFs of patients with hypertrophic cardiomyopathy 42. In this study, we discovered that Meox1 was obviously upregulated in Myofbs after MI. Subsequently, the animal experiment results further indicated that the absence of Meox1 in Myofbs limited post-MI tissue fibrosis, reduced adverse ventricular remodeling, and improved cardiac function. The increased mechanical strain in the marginal zone caused by post-infarction collagen-rich scars may lead to further expansion of fibrotic area, decrease of tissue compliance, and elevation of cardiac afterload, ultimately exacerbating heart failure 25, 43. Our research results showed that Meox1 knockdown could restrict the expansion of scar tissues post-MI and contribute to the alteration of ECM composition in injured heart area, featured as reduced deposition of collagen-rich ECM and more compliant collagen fibers. These findings were consistent with Tani et al.'s scRNA-seq that Meox1 expression was upregulated in CFs in chronic MI mice and cardiac reprogramming induced antifibrotic effects by suppression of Meox1 24.

Myofbs have been identified as the predominant cell type responsible for the production of ECM in post-injury hearts, and the sustained activity of Myofbs results in excessive fibrotic scarring and ultimately pathological fibrosis progressing and cardiac function deteriorating. TGF-β1, the most important signal in fibroblast activation, is significantly upregulated in most heart diseases and plays a major role in cardiac fibrosis 44. Previously, Wei et al. found that TGF-β1 regulated the transcriptional activity of P311 by affecting Meox1 expression, which modulated the proliferation and migration of human dermal fibroblasts 22. Furthermore, in primary fibroblasts of human lungs, livers and kidneys, the expression levels of Meox1 also changed with cell activation status 23. Similarly, we observed increases in the expression and nuclear location of Meox1 in NMCFs upon TGF-β1 stimulation. Furthermore, we discovered that Meox1 knockdown inhibited, whereas overexpression of Meox1 enhanced, TGF-β1-induced CFs-to-Myofbs transformation. Therefore, our *in vivo* and *in vitro* data confirmed that suppressing Meox1 restrained cardiac fibrosis, reduced pathological remodeling, and improved cardiac function through blocking the acquisition of Myofbs phenotype.

While the luciferase reporter assays indicate that Meox1 enhances the transcriptional activity of the Cthrc1 promoter, *in vitro* experiments show that Meox1 overexpression alone is insufficient to induce Cthrc1 protein expression unless TGF-β stimulation is present. This suggests that Meox1-mediated Cthrc1 activation is context-dependent, possibly requiring co-regulatory factors or chromatin remodeling events that are induced only under pathological conditions such as MI or TGF-β-mediated stress. These findings highlight the complexity of transcriptional regulation in fibrosis and the importance of cellular context in Meox1-regulated gene expression. To our knowledge, there are few studies investigating the specific molecular mechanisms by which Meox1 promoted cardiac fibrosis. Here, we performed the bioinformatic analysis to show that Cthrc1, a highly conserved secreted glycosylated protein, may be an important potential target of Meox1. Cthrc1 was initially discovered in a comparative screening for DEGs between balloon-injured and normal rat arteries 45. Subsequent studies showed that Cthrc1 was involved in many physiological and pathological processes, including vascular remodeling, developmental morphogenesis, inflammatory arthritis, bone formation, and tumor progression 46, 47. It has been reported that Cthrc1 is closely related to the development of pathological fibrosis in multiple body tissues and organs and plays different functions in various diseases. For example, administration of exogenous Cthrc1 had both preventive and therapeutic effects on cholestatic liver fibrosis 36. Additionally, Cthrc1 reduced fibrotic tissue formation in bleomycin-induced lung fibrosis 48. However, there was also research report that the absence of Cthrc1 attenuated mouse liver fibrosis induced by carbon tetrachloride or thioacetamide 34. In the porcine MI model, higher levels of Cthrc1 expression, particularly at a later time point, was associated with worse cardiac function and larger infarct areas 28. In this study, we found that the levels of Cthrc1 were increased in both circulating blood and cardiac tissues after MI. Meanwhile, *in vivo* and *in vitro* experiments showed that Cthrc1 was mainly expressed in activated fibroblasts, which was consistent with previous research findings that Myofbs were characterized by a predominant level of Postn and Cthrc1 49. Previous sequencing results by Ruiz et al. demonstrated that the gene expression associated with cell division, proliferation, and ECM proteins synthesis were significantly downregulated in CFs of Cthrc1 knock-out mice after MI 28. Similarly, we observed that silencing of Cthrc1 inhibited proliferation, migration, and ECM proteins synthesis induced by TGF-β1. Moreover, Cthrc1 overexpression in mouse hearts further exacerbated post-MI fibrosis, promoted adverse remodeling, and impaired cardiac function. Our results also supported previous findings from scRNA-seq that Cthrc1-positive pathological fibroblasts expressed the highest levels of collagens in multiple collagen-producing subpopulations and the expansion of this subpopulation contributed to rapidly ensuing pulmonary fibrosis 50, 51. Consequently, given that Cthrc1 is a secreted protein and can be detected in circulating blood, it might serve as a potentially reliable target for diagnosis, monitoring, and therapy in patients with chronic cardiac fibrosis.

Smad2/3, as a canonical downstream signaling molecule of TGF-β, plays a pivotal role in fibroblast activation and the induction of profibrotic gene expression during tissue fibrosis. However, accumulating evidence suggest that the effect of Cthrc1 on TGF-β/Smad signaling is highly cell type- and context-dependent. For example, in smooth muscle cells and keloid fibroblasts, Cthrc1 has been reported to reduce collagen deposition by suppressing TGF-β/Smad pathway 52-54. Additionally, Cthrc1 accelerated the degradation of p-Smad3 via proteasome pathway, thereby inhibiting the synthesis of profibrotic genes 36. In contrast, Li et al. demonstrated that autocrine Cthrc1 derived from hepatic stellate cells facilitated their activation, migration, and contractility by enhancing the activation of Smad2, Smad3, and Smad4 34. Moreover, a recent work by Balazova et al. revealed that Cthrc1 can increase Smad3 phosphorylation via activation of the membrane receptor GPR180 in adipocytes 33. These apparently contradictory findings collectively suggest that the regulatory role of Cthrc1 in TGF-β/Smad signaling is not universal but rather determined by specific cellular and pathological contexts.

In our study, we observed that Cthrc1 overexpression elevated, while its knockdown reduced, the phosphorylation levels of Smad2/3 both *in vitro* and *in vivo*. Notably, Meox1-induced fibroblast activation and p-Smad2/3 upregulation were abolished when Cthrc1 was inhibited, indicating that Cthrc1 functions as an essential mediator of Meox1-driven fibroblast activation. However, our data did not distinguish whether Cthrc1 promoted Smad2/3 phosphorylation through enhancing TGF-β1 production or via a more direct mechanism of signal modulation. Future studies are necessary to elucidate whether Cthrc1 acts on the upstream of TGF-β ligand synthesis, or alternatively, facilitates Smad2/3 activation through receptor-mediated signaling (e.g., via GPR180 or other membrane partners).

In summary, our work reveals a novel molecular mechanism that Meox1 in CFs plays a crucial role in the regulation of post-MI fibrotic remodeling in mice. From the clinical translational perspective, the present study suggests that Meox1/Cthrc1/p-Smad2/3 signaling pathway is a promising prognostic and therapeutic target for post-MI cardiac fibrosis and heart failure.

## Supplementary Material

Supplementary materials and methods, figures, and tables.
