# Supplementary material for: Meox1 Promotes Cardiac Fibrosis and Pathological Remodeling following Myocardial Infarction through Cthrc1/p-Smad2/3 Signaling
Source: Int J Biol Sci. 2026 Jan 1;22(1):410–25. doi: 10.7150/ijbs.113825 (PMC12681941; doi:10.7150/ijbs.113825)
Supplement: Supplementary file 1 — Supplementary materials and methods, figures, and tables. [file ijbsv22p0410s1.pdf]

Meox1 promotes cardiac fibrosis and pathological remodeling following myocardial infarction through Cthrc1/p-Smad2/3 signaling

Mian Zhang[1,2†], Xiao-wen Meng[1,2†], Yu-fan Yang[1,2†], Xin-yu Chen[1,2], Yi-chan Wang[1,2], Jing-jie Wan[1,2], Jun Ding[1,2], Bi-ying Wang[1,2], Ke Peng[1,2*], Fu-hai Ji[1,2*]

[1] Department of Anesthesiology, The First Affiliated Hospital of Soochow University, Suzhou 215006, China

[2] Institute of Anesthesiology, Soochow University, Suzhou 215006, China

[†]These authors contributed equally to this work.

[*]Correspondence to:

Fu-hai Ji, jifuhai@suda.edu.cn, Department of Anesthesiology, The First Affiliated Hospital of Soochow University, Suzhou 215006, China.

Ke Peng, pengke@suda.edu.cn, Department of Anesthesiology, The First Affiliated Hospital of Soochow University, Suzhou 215006, China.

SUPPLEMENTARY MATERIALS

Supplementary methods

1.1 Mouse model of myocardial infarction

The MI model was established by permanent left anterior descending (LAD) artery ligation. The detailed operations of sham and MI surgery were performed as previously described [1, 2]. Briefly, mice were anesthetized by intraperitoneal injection of pentobarbital sodium (50 mg/kg body mass) and then were placed on a heating pad in the supine position to sustain the body temperature within the range of 36.5℃ to 37.5℃. Following endotracheal intubation with a plastic catheter and body weight adjusted tide volume using a small animal ventilator, mice were fixed in a right lateral position for left lateral thoracotomy and the subsequent ligation of LAD artery. A small cutaneous incision was made between the third and fourth intercostalia of the left rib to expose internal chest after confirming sufficient analgesia and surgical tolerance. Under direct visualization with a stereoscopic microscope, the pericardial sac was opened and the LAD artery was located, sutured and ligated with a 6.0 polyfilament silk suture at a point 2 mm away from its origin. Successful ligation and occlusion of the coronary blood flow was visually confirmed by immediate blanching of the anterior wall of the left ventricle as well as by ST segment elevation on electrocardiogram (ECG) monitoring. Subsequently, the pneumothorax was evacuated and the intercostal as well as the skin incisions were closed using a 5.0 suture. Mice were closely monitored until spontaneous breathing occurred, extubated and placed under a heating lamp. Sham operated mice underwent the same surgical procedure, except for LAD artery ligation. In this study, we excluded female mice in *in vivo* experiments in case that estrogen and its receptors might result in biased consequences when analyzing.

1.2 Tissue harvesting and cardiac weight indexes

At the indicated time, mice were weighed and then sacrificed under anesthesia. After blood collection, their hearts were harvested rapidly and washed with cold PBS to remove blood cells. Heart weight (HW) and tibia length (TL) were measured respectively. Then, the ratios of heart weight/body weight (HW/BW, mg/g) and heart weight/tibia length (HW/TL, mg/mm) were calculated to evaluate cardiac hypertrophy.

1.3 Echocardiography analysis

Cardiac structure and function *in vivo* were detected by transthoracic

echocardiography (VisualSonics VeVo 2100 Imaging System, Toronto, Canada) equipped with MS-400 transducer (18–38 MHZ). Briefly, mice were anesthetized by 1.5% isoflurane inhalation and subsequently placed on a heated imaging platform. Four-limb lead ECG was recorded continuously throughout the imaging process and the heart rate was maintained at around 450 bpm. Echocardiographic images were recorded in two-dimensional M-mode from parasternal short axis (PSAX) view at two papillary muscle level. Left ventricular (LV) end-systolic and end diastolic diameters were respectively obtained according to M-mode tracings, including LV posterior wall (LVPW) thicknesses, interventricular septum (IVS) thicknesses, LV internal diameter (LVID). Ejection fraction (EF), fractional shortening (FS), LV internal volume at the end of diastolic (LVEDV) and systolic (LVESV) were calculated automatically by echocardiographic software. The echocardiographic parameters were calculated in three consecutive cardiac cycles and averaged for further analysis.

1.4 Histopathology analysis

The hearts of the mice were excised and fixed in 4% paraformaldehyde for 48 h after perfused with cold phosphatase buffer saline (PBS). Then the hearts were dehydrated, embedded in paraffin, and cut transversely into sections (5 µm thickness). Following dewaxing, the cardiac tissue sections were performed according to the manufacturer's instructions for Masson staining kit (Solarbio, #G1340). The ratio of total scar circumferential length (shown in white and blue in the Masson staining images) to total cardiac cross-sectional circumference was used to estimate the percent circumference of the scar tissue. All circumferential length was measured using ImageJ software (Version 1.6.0, NIH, Bethesda, USA). Picrosirius red (PSR) staining was used to assess collagen fiber deposition and conducted following the manufacturer’s guideline (Servicebio, #G1078). The total fibrotic area in each heart section was quantified using ImageJ software. The proportion of myocardium fibrosis per heart was calculated as fibrotic area divided by LV area. To assess the degree of myocardial hypertrophy, the dewaxed sections were stained with FITC-conjugated wheat germ agglutinin (WGA, Invitrogen, #W11261) for 30 min at 37°C in the dark. After nuclear staining with 4,6-Diamidino-2-phenylindole (DAPI, abcam, #ab104139), the heart sections were photographed using fluorescence microscope (Olympus, Tokyo, Japan) with captured pictures. The cardiomyocyte cross-sectional area was measured by ImageJ software. Immunohistochemistry staining was performed in

deparaffinized sections using a microwave-based antigen retrieval technique as previously described [3]. The Slides were blocked with 5% serum, followed by incubation in rabbit anti-Collagen I antibody (Proteintech, #14695-1-AP) or mouse anti-Periostin antibody (Proteintech, #66491-1-Ig) overnight at 4℃. The sections were incubated with biotinylated secondary antibodies for 1 h at room temperature followed by diaminobenzidine (DAB, Beyotime, P0203) treatment. Afterwards, slides were stained with haematoxylin, dehydrated and imaged under a brightfield microscope.

1.5 Immunofluorescence staining

Immunofluorescence staining of paraffin-embedded heart sections and cultured primary cells was carried out by following standard protocol. Briefly, heart sections were deparaffinized and then subjected to antigen retrieval in a hot citrate solution (Servicebio, #G1202) with microwave oven. The primary cells were fixed in 4% paraformaldehyde for 15 min. The sections and cells were permeabilized with 0.3% Triton-X100 (Servicebio, #GC204003) in PBS for 15 min and then blocked with 5% bovine serum albumin (BSA, Servicebio, #GC305010) in PBS for 1 h at room temperature, followed by incubating with primary antibodies overnight at 4℃ and Alexa Fluor 488- or Alexa Fluor 594-conjugated secondary antibodies at room temperature for 1 h. After washing with PBS, DAPI was used for the nuclear staining. Finally, immunofluorescence staining images were acquired with an immunofluorescence microscope using the same exposure parameters. The detailed information of the primary and secondary antibodies is listed in **Table S1**.

1.6 Isolation of neonatal mice cardiac fibroblasts

Neonatal mice cardiac fibroblasts (NMCFs) were isolated by using the differential time adherent method as we previously described with some modifications [4]. Briefly, 1-3 day old neonatal C57BL/6J mice were executed and their hearts were excised quickly. Subsequently, these hearts were washed with cold PBS to remove blood cells. LV tissues were excised into small pieces and digested in a solution containing 1 mg/ml type II collagenase (Sigma Aldrich, #V900892) at 37℃ for 20 min under constant shaking. After filtering through a 70 μm sterile cell strainer to eliminate tissue debris, the digestion medium containing primary cells was centrifuged at 1500 rpm for 5 min. Then the cells were resuspended in DMEM/F12 (Gibco, #C11330500BT) supplemented with 10 % fetal bovine serum (FBS, Corning, #35-

082-CV) and 1% penicillin/streptomycin (Procell, #PB180120), and then seeded into poly-L-Lysine (Procell, #PB180523) precoated plates. After incubation at 37℃ with 5% CO_2 for 90 min, the supernatant was discarded and the cells adherent to the bottom of plates were NMCFs.

1.7 NMCFs culture and stimulation

NMCFs were cultured with DMEM/F12 medium at 37℃ in an incubator containing 5% CO_2. The medium was changed every 3 days and cells of passage 2 or 3 generation were used for the following experiments. For stimulation, NMCFs were seeded in 6 well plates at day 1. The next day, recombinant transforming growth factor-beta 1 (TGF-β1, Peprotech, #100-21) was added into the medium at a concentration of 10 ng/ml. After 48 h treatments, NMCFs were collected for subsequent analysis.

1.8 siRNA transfection

Specific small interfering RNA (siRNA) targeting Meox1 and negative control siRNA sequence were designed and synthesized by Sangon Biotech Co., Ltd. (Shanghai, China). Target sequence of Cthrc1 siRNA and scramble control siRNA were designed and synthesized by RiboBio Co., Ltd. (Guangzhou, China). The detailed sequence information of Meox1 siRNA and Cthrc1 siRNA was shown in **Table S2**. Transient transfection of siRNA was performed using Lipofectamine® 2000 transfection reagent (Invitrogen, #11668019) following the manufacturer's instructions. Briefly, NMCFs were transfected with 12 nmol/L of siRNA, using 5 μl Lipofectamine 2000 and 1200 μl Opti-MEM (Gibco, #11058021) for each well. At the culmination of 48 h following siRNA transfection, cells were collected and subjected to analysis via qRT-PCR and western blot.

1.9 Plasmid transfection

Expression plasmid encoding mouse Meox1 with a pcDNA3.1 vector were purchased from RiboBio Co., Ltd. (Guangzhou, China). Plasmids were transfected with Lipofectamine 2000 according to the instructions with some modifications. Briefly, 2.5 μg plasmid DNA and 6 μl Lipofectamine 2000 reagent were respectively diluted in 150 μl Opti-MEM. Both solutions were left for 5 min before being combined, then mixed and left for further 20 min. Transfection complex was added to the NMCFs and incubated for 6 h at 37 ℃. Afterwards, the medium was replaced with fresh medium containing 10% FBS, and NMCFs were cultured for another 24 h at 37 ℃ followed by TGF-β1 stimulation.

1.10 AAV construction and delivery

Adeno-associated virus (AAV) carrying a periostin promoter (postn-promoter) driving the expression of shRNA targeting Meox1 (AAV-postn-shMeox1) or negative control (AAV-postn-shNC) was constructed by Hanbio Biotechnology (Shanghai, China). The sequences of the shRNA for shMeox1 were as follows: CGCCAATGAGACGGAGAAGAA and TTCTTCTCCGTCTCATTGGCG. Full-length cDNA encoding mouse Cthrc1 was amplified by PCR and inserted into the vector (purchased from Hanbio Biotechnology Co. Ltd, Shanghai, China), under the control of the mouse cytomegalovirus (CMV) promoter. Virus packaging was performed in HEK293T cells after co-transfection of Cthrc1 or mock vector using Lipofectamine 3000 reagent (Invitrogen, L3000001). After propagating in HEK293T cells, the recombinant AAV underwent purification and tittering. In order to further evaluate the effect of cardiac fibroblasts Meox1 and Cthrc1 in *in vivo* studies, AAV harboring corresponding genes were injected into 5 sites around ischemic border zone of LV (5 μl per site) using a micro-injector following LAD artery ligation.

1.11 ELISA

Cthrc1 levels in serum and cardiac tissue were examined using a commercially available enzyme-linked immunosorbent assay (ELISA) kit (Cloud-Clone Corp., #SEN690Mu). For tissue sample preparation, cardiac tissues were homogenized in fresh ice-cold lysis buffer (Cloud-Clone Corp., #IS007), sonicated with an ultrasonic cell disrupter and then centrifuged for 5 min at 10,000 ×g. The supernatant was collected and used for subsequent assay. The measurement was conducted following the manufacturer's instructions. The absorbance of each well was read at 450 nm with a microplate reader.

1.12 CCK-8 assay

NMCFs were seeded into 96-well plates at a density of 6,000 cells/well and cultured for 24 h, followed by indicated treatment and TGF-β1 stimulation. Cell Counting Kit-8 (CCK-8) (APExBIO, #K1018) were used to detect the cell proliferative viability at corresponding time points. In brief, the mixed solution containing 10 μl CCK-8 reagent and 90 μl DMEM/F12 medium was added to each well and incubated for 3 h at 37 ℃. The absorbance was measured at 450 nm using a microplate reader.

1.13 EdU assay

5-Ethynyl-2’-Deoxyuridine (EdU) staining assay was used for assessing cell

proliferation ability. After TGF-β1 treatment, NMCFs were co-incubated with EdU working solution (APExBIO, #K1075) for a 6 h period under 37 ℃ and 5%CO_2. Subsequently, the cells were fixed, permeabilized and incubated with click reaction solution at room temperature for EdU fluorescence labeling. Besides, cell nuclei were dyed with Hoechst solution in dark. The proliferating cells were stained red, whereas the nuclei of all cells were stained blue. Images were captured using an inverted fluorescence microscope. Cell counting was performed by ImageJ software. The percentage of EdU positive cells was calculated by the following formula: EdU positive cell (%) = EdU positive cell count/Hoechst positive cell count × 100%.

1.14 Scratch wound healing assay

Cell migration was detected by a scratch wound healing assay. In brief, NMCFs were seeded in 6-well cell culture plates. A straight scratch was made on the cell monolayer of each well using a sterile pipette tip after indicated treatments. The detached cells were removed by washing with PBS. Then, the medium was replaced with fresh DMEM/F12 medium containing TGF-β1 (10 ng/ml) or solvent. The scratch areas were immediately photographed post-scratching (0 h) under a light microscope. Following 48 h incubation at 37 ℃ in a CO_2 incubator, images of scratch wound were separately captured again. The areas of each scratch wound at 0 h and 48 h were measured respectively, using OlyVIA software (Olympus, Tokyo, Japan). Cell migration ability was evaluated by calculating the recovery of the scratch area, which was expressed as the percentage of the closed scratch area to the initial scratch area.

1.15 RT-qPCR

Total RNA was extracted from cultured cells using Trizol reagent (Invitrogen, #15596026CN) according to the manufacturer’s specifications. The concentration and quality of RNA were detected using a Nanodrop spectrophotometer (Thermo Fisher Scientific, Waltham, USA). Then, 700 ng of total RNA from each sample, possessing an A260/A280 ratio within the range of 1.8-2.0, was reverse-transcribed to complementary DNA (cDNA) using the HiScript III RT SuperMix for qPCR (Vazyme, #R323) following the manufacturer’s guidelines. Real-Time quantitative polymerase chain reaction (RT-qPCR) was performed using the AceQ Universal SYBR qPCR Master Mix (Vazyme, #Q511) in a LightCycler 480 instrument (Roche, Basel, Switzerland). The total volume of the reaction system was 10 μl, including 5 μl of SYBR qPCR Master Mix, 0.4 μl of forward primers, 0.4 μl of reverse primers, 1 μl of

cDNA and 3.2 μl of ddH_2O. The specific primer sequences used in RT-qPCR were shown in **Table S3**. The thermal cycle program was set as follows: 95 ℃ for 5 min (initial denaturing step), followed by 95 ℃ for 10 s and 60 ℃ for 30 s (40 cycles). All samples were run in duplicates. The relative quantitation of gene expression was analyzed by using the standard $2^{-\Delta\Delta CT}$ method and glyceraldehyde-3-phosphate dehydrogenase (GAPDH) served as the internal control gene.

1.16 Western blot

Total proteins were extracted from cardiac tissues or cultured cells using RIPA lysis buffer (Beyotime, #WB3100) supplemented with protease inhibitor cocktail (Beyotime, #P1010) and phosphatase inhibitor cocktail (APExBIO, #K1012) according to the manufacturer's instructions. The concentration of the sample proteins was measured with a bicinchoninic acid (BCA) protein assay kit (Beyotime, #P0021). Equal amounts of protein extracts were added to sodium dodecyl sulfate polyacrylamide gels and separated by electrophoresis. Subsequently, the proteins in the gels were transferred to polyvinylidene fluoride (PVDF) membranes (Merck, #ISEQ00010), followed by blocking with 5% defatted milk in tris buffered saline with tween (TBST) for 2 h at room temperature. The PVDF membranes were separately incubated with indicated primary antibodies overnight at 4℃. After washing with TBST, the membranes were individually incubated with horseradish peroxidase (HRP)-conjugated goat anti-mouse (Fdbio Science, #FDM007) or goat anti-rabbit (Fdbio Science, #FDR007) secondary antibodies for 90 min at room temperature. Eventually, the specific protein blots were visualized with an enhanced chemiluminescence (ECL) detection substrate (APExBIO, #K1231), and the images were captured by a chemiluminescence detection system (Tanon, China). The densities of the protein blots were quantified using ImageJ software and the relative expression level of target protein was normalized against GAPDH. The detailed information about primary antibodies used in western blot were listed in **Table S1**.

1.17 Differential expression analysis

To provide further validation of our model, we used publicly available gene expression datasets (GSE110209, GSE186079, GSE202228). The R software package was used to process and identify the differentially expressed genes (DEGs) in each dataset analysis. The *P*-values calculated by t-test were then corrected for multiple comparisons using the false discovery rate method. The cutoff threshold for DEG was

set to an absolute value of fold change >2 and a *P*-value <0.05.

1.18 Gene enrichment analysis

For the functional enrichment analysis of the common DEGs in GSE110209 and GSE186079, gene ontology (GO) annotations of genes from the R software package "org.Hs.eg.db" (version 3.1.0) were used as background. The selected genes were mapped to the background set, and the gene enrichment analysis was conducted using the R software package "ClusterProfiler" (version 3.14.3). Moreover, the latest Kyoto encyclopedia of genes and genomes (KEGG) pathway was acquired from the KEGG application programming interface (API). The top 20 enriched terms under each category were selected for plotting and eventually visualized as bar plots.

1.19 Luciferase reporter assays

For the construction of Cthrc1 promoter luciferase reporter vector, a fragment containing the full-length mouse Cthrc1 promoter sequence was amplified by PCR, then synthesized and cloned into the GPL4-Basic luciferase reporter vector between the NheI and HindIII loci (purchased from GenePharma Co., Ltd., Suzhou, China). For investigation of the effect of Meox1 overexpression on Cthrc1 promoter, HEK293T cells in 24-well plates were co-transfected with Cthrc1 promoter luciferase reporter plasmid and Meox1 overexpression plasmid (OE-Meox1) using Lipofectamine 2000 transfection reagent. The Renilla luciferase reporter plasmid (pRL-TK, Promega) was used as a control for transfection efficiency. 48 h after transfection, cells were harvested and the luciferase activity was detected using a dual luciferase reporter assay system (Promega, Madison, USA) according to the manufacturer's protocol. Firefly luciferase activity was normalized to the internal transfection control provided by Renilla luciferase activity. The normalized relative light unit (RLU) values obtained from cells treated with GPL4-control vector and vehicle were considered one-fold induction, from which the activity induced by the various treatments was calculated. Each experiment contained three replicates and was repeated three times.

1.20 Distinct cardiac cell populations isolation

The isolation of distinct cardiac cell populations (cardiomyocytes, endothelial cells and fibroblasts) from the ventricles of male mice was performed 28 days following operation. This was done as previously described with some modifications [5]. Briefly, mice were anesthetized, and the chest was opened to exposed the heart. Then 7 ml

EDTA buffer (130 mM NaCl, 5 mM KCl, 0.5 mM Na_2HPO_4, 10 mM HEPES, 10 mM Taurine, 10 mM D-glucose, 10 mmol/l BDM, 5mM EDTA, pH 7.8) was injected steadily into the apex of right ventricle after cutting the descending aorta and inferior vena cava. The heart was then excised and transferred to a sterile dish containing fresh EDTA buffer after clamping ascending aorta. Digestion was achieved by sequential injection of 10 ml EDTA buffer, 3 ml perfusion buffer (130 mM NaCl, 5 mM KCl, 0.5 mM Na_2HPO_4, 10 mM HEPES, 10 mM Taurine, 10 mM D-glucose, 10 mmol/l BDM, 1mM $MgCl_2$, pH 7.8), and 30 to 50 ml collagenase buffer (perfusion buffer with 0.5 mg/ml collagenase II, 0.5 mg/ml collagenase IV and 0.05 mg/ml protease XIV) into the apex of left ventricle. Subsequently, left ventricle was separated and gently pulled into small pieces in collagenase buffer. Cellular digestion was inhibited by addition of 5 ml stop buffer (comprising perfusion buffer with 5% FBS). The cell suspension was passed through a 100-μm filter into a 50 ml tube, allowing the formation of a pellet through gravity settling. The pellet with CMs underwent 3 steps of calcium reintroduction and were plated on laminin (5 μg/ml) precoated culture dishes. The remaining cellular components was collected and then filtrated through 70 μm strainers to obtain non-cardiomyocyte fraction. The supernatant was incubated with CD31 MicroBeads (Miltenyi Biotec, #130-097-418) (1×10^7 total cells per 10 μl MicroBeads) for 15 minutes at 4 ℃ and purified through the LD Column (Miltenyi Biotec, #130-042-901) according to the manufacturer’s instructions. The magnetically labeled cells were endothelial cells ($CD31^+$), and the unlabeled cells that passed through the column were CFs ($CD31^-$).

1.21 Statistical Analysis

Statistical analyses were performed with GraphPad software (GraphPad Prism, version 8.0, La Jolla, USA). All quantitative data were presented as mean ± standard deviation (SD). The unpaired, two-tailed Student's *t*-test was used to analyze the difference between the two groups with normally distributed data. One-way analysis of variance (ANOVA) followed by Dunnett's or Tukey's post-hoc test was used for comparisons between multiple groups when normal distribution was satisfied. Survival analysis was performed by the Kaplan–Meier method, and the outcomes were subsequently compared using the log-rank test. Statistical significance was set at *p* values less than 0.05.

References

1. Sun P, Wang C, Mang G, Xu X, Fu S, Chen J, et al. Extracellular vesicle-packaged mitochondrial disturbing miRNA exacerbates cardiac injury during acute myocardial infarction. Clin Transl Med. 2022; 12: e779.
2. Huang G, Cheng Z, Hildebrand A, Wang C, Cimini M, Roy R, et al. Diabetes impairs cardioprotective function of endothelial progenitor cell-derived extracellular vesicles via H3K9Ac inhibition. Theranostics. 2022; 12: 4415-30.
3. Park S, Baek IJ, Ryu JH, Chun CH, Jin EJ. PPARalpha-ACOT12 axis is responsible for maintaining cartilage homeostasis through modulating de novo lipogenesis. Nat Commun. 2022; 13: 3.
4. Zhang M, Lei YS, Meng XW, Liu HY, Li LG, Zhang J, et al. Iguratimod Alleviates Myocardial Ischemia/Reperfusion Injury Through Inhibiting Inflammatory Response Induced by Cardiac Fibroblast Pyroptosis via COX2/NLRP3 Signaling Pathway. Front Cell Dev Biol. 2021; 9: 746317.
5. Ackers-Johnson M, Li PY, Holmes AP, O'Brien SM, Pavlovic D, Foo RS. A Simplified, Langendorff-Free Method for Concomitant Isolation of Viable Cardiac Myocytes and Nonmyocytes From the Adult Mouse Heart. Circ Res. 2016; 119: 909-20.

Supplementary tables

2.1 Table S1. Antibodies used in this study.

Target antigen	Vendor or Source	Cat. No.	Application	Dilution
GAPDH	Fdbio Science	#FD0063	WB	1:40000
Collagen I	Proteintech	#14695-1-AP	WB	1:1500
Postn	Proteintech	#66491-1-Ig	WB	1:500
MMP2	ZenBio	#R24993	WB	1:1000
Vimentin	Abways	#CY5134	WB, IF	1:35000 (WB)<br>1:100 (IF)
TGF-β1	ZenBio	#346599	WB	1:1000
α-SMA	ZenBio	#380653	WB, IF	1:35000 (WB)<br>1:100 (IF)
Cthrc1	Proteintech	#16534-1-AP	WB, IF	1:1000 (WB)<br>1:50 (IF)
Meox1	Invitrogn	#PA5-21037	WB, IF	1:1000 (WB)<br>1:50 (IF)
Fibronectin-1	ZenBio	#250073	WB	1:500
α-actinin	Santa Cruz	#sc-17829	IF	1:50
CD31	Abcam	#ab222783	IF	1:50
Smad2/3	ZenBio	#382472	WB	1:1000
p-Smad2/3	ZenBio	#251795	WB	1:500
p-Smad2/3	Abclonal	#AP0548	IF	1:50
Goat Anti-Rabbit IgG H&L (Alexa Fluor® 488)	abcam	# ab150077	IF	1:500
Goat Anti-Rabbit IgG H&L (Alexa Fluor® 594)	abcam	#ab150080	IF	1:200
Goat Anti-Mouse IgG H&L (Alexa Fluor® 488)	abcam	# ab150113	IF	1:500
HRP-labeled Goat Anti-Rabbit IgG(H+L)	Fdbio	# FDR007	WB	1:5000
HRP-labeled Goat Anti-Mouse IgG(H+L)	Fdbio	# FDM007	WB	1:5000

2.2 Table S2. Detailed sequence information of Meox1 siRNA and Cthrc1 siRNA used in this study.

siRNA name	Sequence (5' to 3')
siMeox1-01	CGAAUCUUCAACGAGCAGCAUTT
siMeox1-02	CGCCAAUGAGACGGAGAAGAATT
siMeox1-03	GAGGAUGAAGUGGAAACGUGUTT
siCthrc1-01	TCGCATCATCATTGAAGAA
siCthrc1-02	TTGAAGAACTACCGAAATA
siCthrc1-03	CGCTGGTATTTTACATTTA

2.3 Table S3. Primer sequence for RT-qPCR.

Gene name	Forward primer (5' to 3')	Reserve primer (5' to 3')
Collagen I	TGAACGTGGTGTACAAGGTC	CCATCTTTACCAGGAGAACCAT
α-SMA	CGTGGCTATTCCTTCGTGACTACTG	CGTCAGGCAGTTCGTAGCTCTTC
Meox1	CGGAGATATGAGATTGCAGTCA	CTTCACACGTTTCCACTTCATC
GAPDH	GGTTGTCTCCTGCGACTTCA	TGGTCCAGGGTTTCTTACTCC
Cthrc1	CGGCAGAGGGAGGTGGTAGAC	CCGAAGCGAGCCACTGAACAG
Fibronectin-1	CTATAGGATTGGAGACACGTGG	CTGAAGCACTTTGTAGAGCATG
Postn	AAGGGAATGACTAGCGAAGAAA	CTCATTCACTAGAAGCGTTTCG
LOX	GAGGTTGGCGAACAAGAGGGAAG	GTAAGGTCACAGCGGTCTCGTTG

2.4 Table S4. Biometric and echocardiographic parameters of mice in sham and MI group at different time point.

Time point	BL		7D		14D		28D	
**Group**	**sham**	**MI**	**sham**	**MI**	**sham**	**MI**	**sham**	**MI**
**BW (g)**	-	-	-	26.769±0.842	-	27.020±1.614	28.324±0.443	29.242±1.110
**TL (mm)**	-	-	-	16.840±0.446	-	17.148±0.506	17.400±0.333	17.216±0.279
**HW (mg)**	-	-	-	128.080±3.982	-	133.360±9.465	118.380±5.874	157.000±10.717
**HW/BW (mg/g)**	-	-	-	4.788±0.166	-	4.944±0.344	4.178±0.174	5.372±0.368
**HW/TL (mg/mm)**	-	-	-	7.606±0.160	-	7.781±0.565	6.803±0.299	9.123±0.664
**LVID, s (mm)**	1.236±0.167	1.546±0.375	1.822±0.374	2.382±0.211	1.528±0.311	2.420±0.258	2.061±0.196	2.726±0.391
**LVID, d (mm)**	2.427±0.294	2.855±0.397	3.172±0.494	3.391±0.276	2.692±0.510	3.191±0.357	3.350±0.244	3.335±0.482
**IVS, s (mm)**	1.486±0.159	1.406±0.193	1.565±0.250	1.603±0.188	1.482±0.217	1.304±0.180	1.641±0.257	1.323±0.147
**IVS, d (mm)**	0.960±0.081	0.975±0.234	0.994±0.198	1.308±0.166	1.062±0.190	0.953±0.231	1.112±0.208	1.130±0.148
**LVPW, s (mm)**	1.369±0.296	1.535±0.303	1.444±0.355	1.369±0.347	1.278±0.242	1.134±0.391	1.671±0.361	1.357±0.297
**LVPW, d (mm)**	1.153±0.108	1.100±0.303	1.074±0.359	1.036±0.451	0.968±0.117	1.051±0.281	1.128±0.297	1.301±0.291
**LVEF (%)**	82.457±1.991	78.775±7.005	75.128±5.718	58.091±1.890	75.381±6.279	49.315±2.518	70.085±2.099	38.772±4.336
**LVFS (%)**	49.122±2.072	46.435±6.822	42.998±4.980	29.804±1.159	42.916±6.596	24.118±1.557	38.581±1.529	18.220±2.321

Data were presented as mean ± SD. Data was analyzed using unpaired Student's *t*-test.

**p*<0.05, ***p*<0.01, ****p*<0.001, MI vs. sham.

BW (g) = body weight;

TL (mm) = tibia length;

HW (mg) = heart weight;

HW/BW (mg/g) = heart weight/body weight;

HW/TL (mg/mm) = heart weight/tibia length;

LVID,s (mm) = left ventricular internal diameter at end-systole;

LVID,d (mm) = left ventricular internal diameter at end-diastole;

IVS,s (mm) = interventricular septum thickness at systole;

IVS,d (mm) = interventricular septum thickness at diastole;

LVPW,s (mm) = left ventricular posterior wall thickness at systole;

LVPW,d (mm) = left ventricular posterior wall thickness at diastole;

LVEF (%) = left ventricular ejection fraction;

LVFS (%) = left Ventricular fractional shortening.

2.5 Table S5. GEO Datasets used for gene differential expression analysis.

GEO	Genus	Sample Type	Control	Heart disease
GSE186079	Mus musculus	Mouse cardiac fibroblasts isolated from left ventricle	sham: n=2<br>GSM5631592-GSM5631593	MI: n=2<br>GSM5631600-GSM5631601
GSE110209	Mus musculus	Left ventricle	sham: n=3<br>GSM2982705-GSM2982707	MI: n=3<br>GSM2982723-GSM2982725
GSE202228	Rattus norvegicus	Left ventricle	sham: n=4<br>GSM6106721-GSM6106724	MI: n=4<br>GSM6106725-GSM6106728
GSE46224	Homo sapiens	Left ventricle	Non-failing: n=8<br>GSM1126612-GSM1126619	Ischemic cardiomyopathy: n=8<br>GSM1126620-GSM1126627

2.6 Table S6. Biometric and echocardiographic parameters of AAV-postn-shNC and AAV-postn-shMeox1 treated mice.

Group	sham+AAV-postn-shNC		sham+AAV-postn-shMeox1		MI+AAV-postn-shNC		MI+AAV-postn-shMeox1	
Time point	BL	28D	BL	28D	BL	28D	BL	28D
BW (g)	-	24.690±1.683	-	25.224±0.975	-	24.790±1.896	-	25.757±1.127
TL (mm)	-	17.333±0.182	-	17.313±0.393	-	17.187±0.215	-	17.316±0.307
HW (mg)	-	110.133±10.576	-	114.633±10.529	-	143.656±4.316	-	125.633±7.460
HW/BW (mg/g)	-	4.462±0.324	-	4.622±0.223	-	5.699±0.141	-	4.881±0.280
HW/TL (mg/mm)	-	6.352±0.592	-	6.611±0.482	-	8.358±0.213	-	7.253±0.364
LVID, s (mm)	1.611±0.222	1.948±0.351	1.578±0.253	1.614±0.340	1.676±0.428	3.348±0.290	1.390±0.261	2.742±0.262
LVID, d (mm)	2.956±0.492	3.257±0.440	2.790±0.397	2.755±0.642	3.092±0.470	3.954±0.344	2.489±0.334	3.665±0.322
IVS, s (mm)	1.488±0.187	1.287±0.156	1.569±0.228	1.053±0.203	1.449±0.179	1.073±0.356	1.572±0.173	1.455±0.192
IVS, d (mm)	1.027±0.242	0.839±0.106	1.125±0.162	1.494±0.220	0.988±0.138	0.888±0.254	1.112±0.150	1.060±0.101
LVPW, s (mm)	1.562±0.307	1.520±0.227	1.510±0.305	1.429±0.328	1.478±0.276	0.995±0.258	1.594±0.251	1.406±0.202
LVPW, d (mm)	1.238±0.307	1.108±0.272	1.225±0.226	1.254±0.309	1.112±0.290	0.937±0.253	1.335±0.167	1.222±0.232
LVEF (%)	76.960±6.920	72.288±4.739	76.191±4.540	73.387±4.922	78.826±6.531	32.794±3.850	77.360±5.488	50.692±2.120
LVFS (%)	44.790±7.328	40.463±3.876	43.473±4.226	40.917±4.573	46.639±6.248	15.303±2.044	44.405±5.517	25.241±1.219

2.7 Table S7. The list of common DEGs between GSE186079 and GSE110209.

	**Gene symbol**
**Common DEGs**	Vdr, Ptn, Capn6, Col11a1, Fmod, Sfrp2, Ltbp2, Pdgfrl, C1qtnf3, Crlf1, Mfap4, C1qtnf6, Col12a1, Col8a1, Cthrc1, Cilp, Fam180a, Tnc, Clec11a, Cercam, Ptgis, Postn, Nkd2, Col1a2, Piezo2, Comp, Cst3, Thbs4, P4ha3, Ssc5d, Ncam1, Col22a1, Col16a1, Col3a1, Lox, Fbln1, Bgn, Cyp26a1, Timp1, Loxl1, Tmem119, Thbs1, Tgfb3, Kcnj15, Frzb, Aebp1, Col8a2, Gpr176, Col5a2, Dkk3, Cilp2, Ddah1, Spp1, Fn1, Col1a1, Acaa2, Enpp1, Rtn2, Ass1, Itgbl1, Zfp185, Meox1, Gpr39, Cyth4, Lhfpl2, Eln, Emilin1, H2-Ab1, Loxl3, Nox4, Csrp2, Fxyd6, Chpf, Ctsk, Map6, Ms4a7, Runx1, Prelp, Endod1, Atp6v0a4, Angptl7, Boc, Dpysl3, Lgals3

2.8 Table S8. Biometric and echocardiographic parameters of MI mice injected with AAV-postn-shNC or AAV-postn-shMeox1 and AAV-NC or AAV-Cthrc1.

Group	MI+AAV-postn-shNC+AAV-NC		MI+AAV-postn-shNC+AAV-Cthrc1		MI+AAV-postn-shMeox1+AAV-NC		MI+AAV-postn-shMeox1+AAV-Cthrc1	
**Time point**	**BL**	**28D**	**BL**	**28D**	**BL**	**28D**	**BL**	**28D**
**BW (g)**	-	24.907±1.799	-	24.290±1.670	-	24.724±2.106	-	24.140±1.791
**TL (mm)**	-	16.897±0.538	-	16.673±0.342	-	16.740±0.420	-	16.660±0.379
**HW (mg)**	-	140.667±8.188	-	146.100±4.353	-	129.850±7.843	-	136.650±6.041
**HW/BW (mg/g)**	-	5.655±0.136	-	6.033±0.287	-	5.263±0.139	-	5.676±0.242
**HW/TL (mg/mm)**	-	8.319±0.243	-	8.761±0.109	-	7.750±0.278	-	8.199±0.206
**LVID, s (mm)**	2.220±0.315	3.416±0.490	2.087±0.360	4.302±1.015	2.060±0.247	3.023±0.167	2.121±0.232	3.870±0.692
**LVID, d (mm)**	3.688±0.457	4.203±0.572	3.433±0.474	4.983±1.059	3.455±0.376	4.081±0.152	3.516±0.391	4.646±0.719
**IVS, s (mm)**	1.456±0.117	0.975±0.271	1.395±0.149	0.725±0.129	1.334±0.178	1.251±0.199	1.323±0.160	0.980±0.206
**IVS, d (mm)**	0.952±0.115	0.770±0.182	0.991±0.144	0.687±0.167	0.9469±0.132	0.930±0.129	0.997±0.058	0.809±0.170
**LVPW, s (mm)**	1.107±0.184	1.036±0.169	1.174±0.065	1.141±0.358	1.168±0.084	1.069±0.156	1.126±0.235	1.235±0.498
**LVPW, d (mm)**	0.748±0.134	0.786±0.188	0.781±0.105	0.969±0.305	0.731±0.098	0.797±0.156	0.892±0.165	0.919±0.502
**LVEF (%)**	71.167±3.956	39.162±1.774	71.036±3.826	29.876±6.899	71.839±5.262	51.342±4.228	71.056±3.674	35.676±5.163
**LVFS (%)**	39.841±3.370	18.774±0.845	39.486±2.844	14.082±3.388	40.335±4.614	25.933±2.624	39.591±3.132	17.045±2.561

2.9 Table S9. Biometric and echocardiographic parameters of MI mice injected with AAV-postn-NC or AAV-postn-Meox1 and AAV-shNC or AAV-shCthrc1.

Group	MI+AAV-postn-NC+AAV-shNC		MI+AAV-postn-NC+AAV-shCthrc1		MI+AAV-postn-Meox1+AAV-shNC		MI+AAV-postn-Meox1+AAV-shCthrc1	
**Time point**	**BL**	**28D**	**BL**	**28D**	**BL**	**28D**	**BL**	**28D**
**BW (g)**	-	24.000±1.176	-	23.750±0.591	-	23.967±0.816	-	23.7±0.572
**TL (mm)**	-	16.747±0.387	-	16.613±0.317	-	16.743±0.249	-	16.767±0.24
**HW (mg)**	-	137.983±4.433	-	129.6±5.504	-	144.35±5.52	-	133.667±4.150
**HW/BW (mg/g)**	-	5.755±0.124	-	5.455±0.137	-	6.023±0.125	-	5.641±0.148
**HW/TL (mg/mm)**	-	8.238±0.124	-	7.798±0.193	-	8.619±0.224	-	7.972±0.195
**LVID, s (mm)**	2.010±0.227	3.184±0.091	2.082±0.190	2.774±0.172	2.176±0.239	3.832±0.550	2.154±0.175	2.724±0.235
**LVID, d (mm)**	3.472±0.249	4.003±0.115	3.638±0.222	3.743±0.174	3.582±0.350	4.496±0.648	3.511±0.264	3.522±0.224
**IVS, s (mm)**	1.318±0.219	1.024±0.074	1.346±0.096	1.130±0.246	1.373±0.115	0.880±0.280	1.357±0.158	1.108±0.088
**IVS, d (mm)**	0.880±0.130	0.963±0.064	0.820±0.135	0.952±0.087	0.880±0.074	0.781±0.192	0.786±0.117	0.952±0.088
**LVPW, s (mm)**	1.290±0.232	1.451±0.113	1.412±0.198	1.362±0.151	1.423±0.124	1.002±0.233	1.235±0.195	1.307±0.155
**LVPW, d (mm)**	0.764±0.119	1.024±0.111	0.975±0.270	1.036±0.068	0.958±0.090	0.886±0.230	0.830±0.113	0.963±0.102
**LVEF (%)**	73.933±5.242	42.235±3.050	74.604±3.860	51.677±3.393	70.584±3.585	31.434±4.601	69.978±2.260	46.616±3.674
**LVFS (%)**	42.167±4.623	20.449±1.754	42.789±3.491	25.929±2.065	39.253±2.978	14.765±2.370	38.641±1.839	22.738±2.074

Supplementary figures

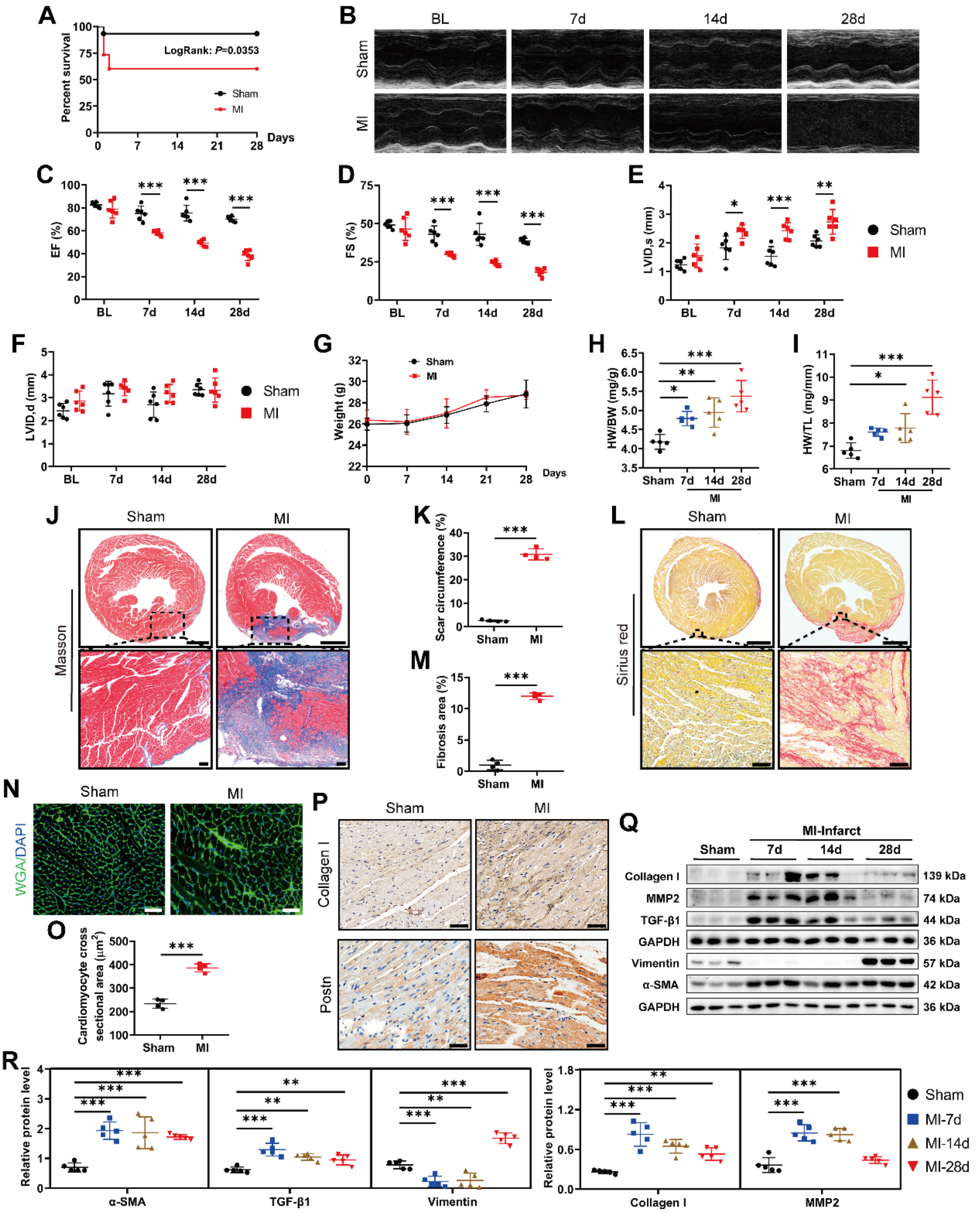

Figure S1. MI induced cardiac dysfunction, adverse remodeling, and cardiac fibrosis. (A) Kaplan-Meier survival curves within 28 days following MI or sham procedures. n=15. **(B)** Representative images of M-mode echocardiography of mice at baseline and on days 7, 14, and 28 in the sham and MI groups. **(C-F)** LVEF, LVFS, LVID,s, and LVID,d. n=6. **(G)** Body weight in the sham and MI groups at different time points. n=5. **(H, I)** The ratios of HW to BW and HW to TL at indicated time points. n=5. **(J, K)** Representative images and quantification of scar circumference of

heart sections stained with Masson trichrome on day 28. Scale bars, 1000 μm (upper) and 100 μm (lower). n=4. **(L, M)** Representative images and quantification of fibrotic area of heart sections stained with Picrosirius red on day 28. Scale bars, 1000 μm (upper) and 50 μm (lower). n=4. **(N, O)** Representative images and quantification of cross-sectional area of cardiomyocyte in heart sections stained with WGA (green) and DAPI (blue) on day 28. Scale bar, 50 μm. n=4. **(P)** Representative images of immunohistochemical staining for Collagen I and Postn in heart sections. Scale bar, 50 μm. **(Q, R)** Representative immunoblot images and quantitative protein levels of α-SMA, TGF-β1, Vimentin, Collagen I, and MMP2 in infarct heart tissues at indicated time points. n=5. Data are mean ± SD. Log-rank test **(A)**, unpaired student's test **(C-G, K, M, O)** and one-way ANOVA followed by Dunnett post hoc test **(H, I, R)**. **P*<0.05, ***P*<0.01, ****P*<0.001.

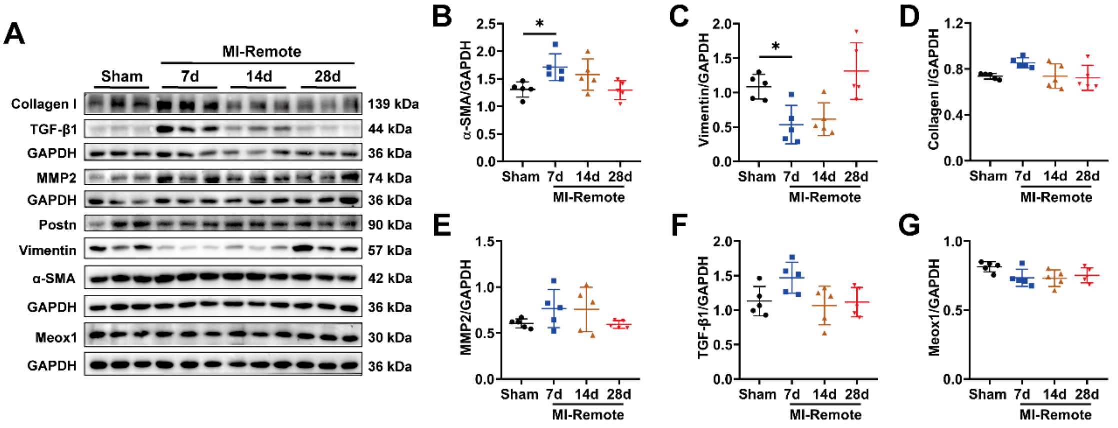

Figure S2. Protein levels of fibrosis-related genes in remote zones of infarct hearts after MI in mice. (A-G) Representative immunoblot images and quantitative analyses of α-SMA, Vimentin, Collagen I, MMP2, TGF-β1 and Meox1 protein levels in the remote zone of infarct hearts at indicated time points. n=5. Data are mean ± SD. **P*<0.05 by one-way ANOVA followed by Dunnett post hoc test.

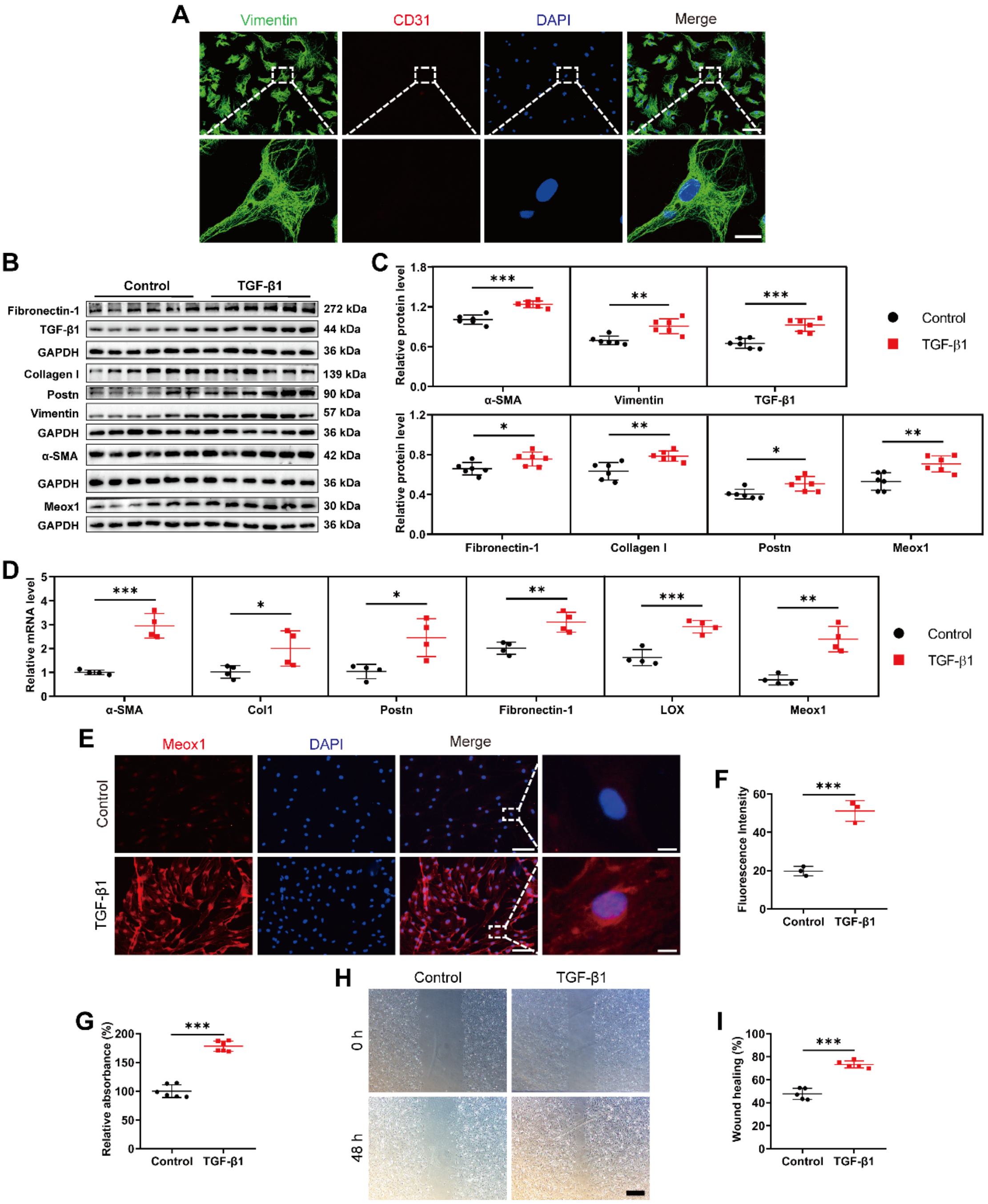

Figure S3. Meox1 was upregulated in activated NMCFs upon TGF-β1 stimulation. (A) Representative micrographs of immunofluorescence staining with Vimentin (green), CD31 (red) and DAPI (blue) in isolated primary cells for the identification of NMCFs. Scale bars, 100 μm (upper) and 50 μm (lower). **(B, C)** Representative immunoblot images and quantitative analyses of α-SMA, Vimentin, TGF-β1, Fibronectin-1, Collagen I, Postn and Meox1 protein levels in NMCFs after treatment with vehicle or TGF-β1. n=6. **(D)** Quantitative analyses of α-SMA, Col1, Postn, Fibronectin-1, Lox and Meox1 mRNA levels in NMCFs. n=4. **(E, F)** Representative immunofluorescence micrographs and quantification of Meox1

fluorescence intensity in NMCFs. Scale bars, 100 μm (left) and 10 μm (right). n=3. **(G)** Proliferation of NMCFs determined by CCK-8 assay. n=6. **(H, I)** Representative scratch micrographs of NMCFs at 0 and 48 h after treatment with vehicle or TGF-β1 and quantification of the area of scratch wound closure. Scale bar, 500 μm. n=5. Data are mean ± SD. **P*<0.05, ***P*<0.01, ****P*<0.001 by unpaired student's test.

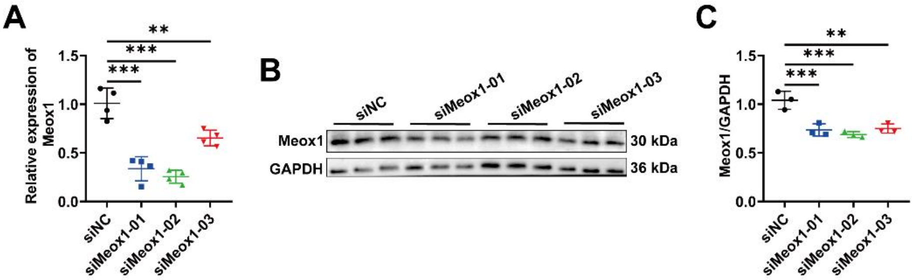

Figure S4. The knockdown efficacy of Meox1 siRNA in NMCFs. (A) RT-qPCR analysis of Meox1 mRNA level in NMCFs transfected with three independent siMeox1 or siNC. n=4. **(B, C)** Representative immunoblot images and quantitative analysis of Meox1 protein level in NMCFs. n=3. Data are mean ± SD. ***P*<0.01, ****P*<0.001 by one-way ANOVA followed by Dunnett post hoc test.

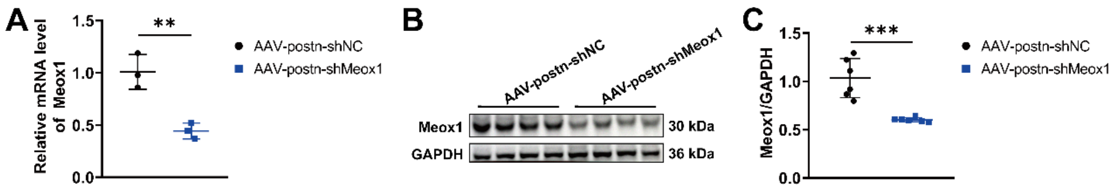

Figure S5. Validation of Meox1 knockdown in *vivo*. (A) RT-qPCR analysis of Meox1 mRNA level in fibroblasts isolated from hearts of adult mice subjected to MI surgery for 28 days. n=3. **(B, C)** Representative immunoblot images and quantitative analysis of Meox1 protein level in fibroblasts isolated from hearts of adult mice subjected to MI surgery for 28 days. n=6. Data are mean ± SD. ***P*<0.01, ****P*<0.001 by unpaired student's test.

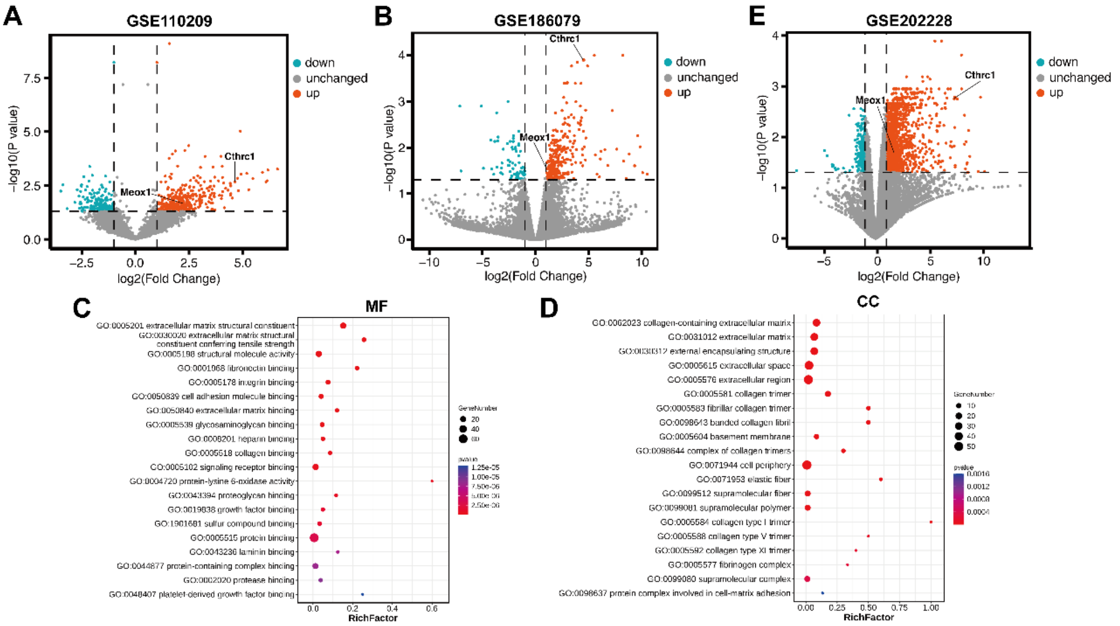

Figure S6. Meox1 and Cthrc1 were highly expressed in the MI animal model and cardiac fibroblasts. (A) The volcano plot showing the DEGs in left ventricular tissues from the sham and MI mice (GSE110209 dataset). **(B)** The volcano plot showing the DEGs in CFs isolated from left ventricular tissues of the sham and MI mice (GSE186079 dataset). **(C, D)** Top 20 molecular function (MF) and cellular component (CC) categories in the gene ontology study of the common DEGs between GSE110209 and GSE186079 datasets. **(E)** The volcano plot showing the DEGs in left ventricular tissues from the sham and MI rats (GSE202228 dataset).

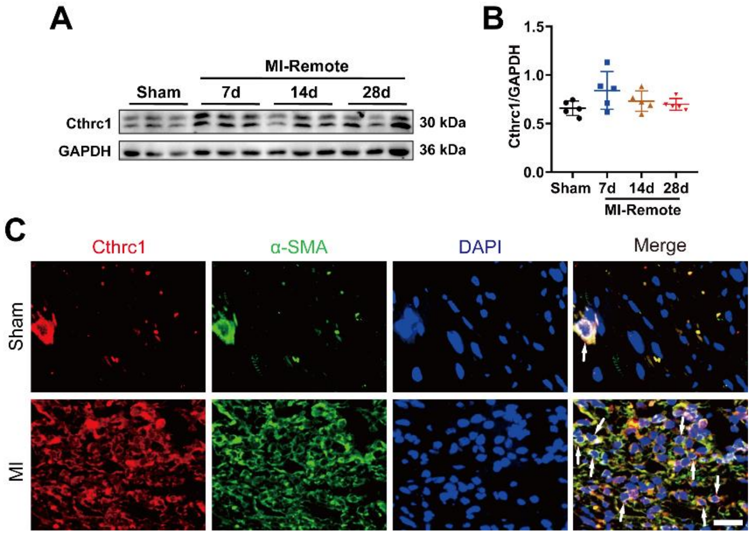

Figure S7. Cthrc1 expression was upregulated in Myofbs of infarct zone following MI. (A, B) Representative immunoblot images and quantitative analysis of Cthrc1 protein level in the remote zone of infarct hearts at indicated time points. n=5. **(C)** Representative immunofluorescence micrographs of Cthrc1 (red), α-SMA (green) and DAPI (blue) in heart tissues at day 28. Arrows indicate representative co-localizations of Cthrc1 and α-SMA. Scale bar, 20 μm. Data are mean ± SD. One-way ANOVA followed by Dunnett post hoc test was used.

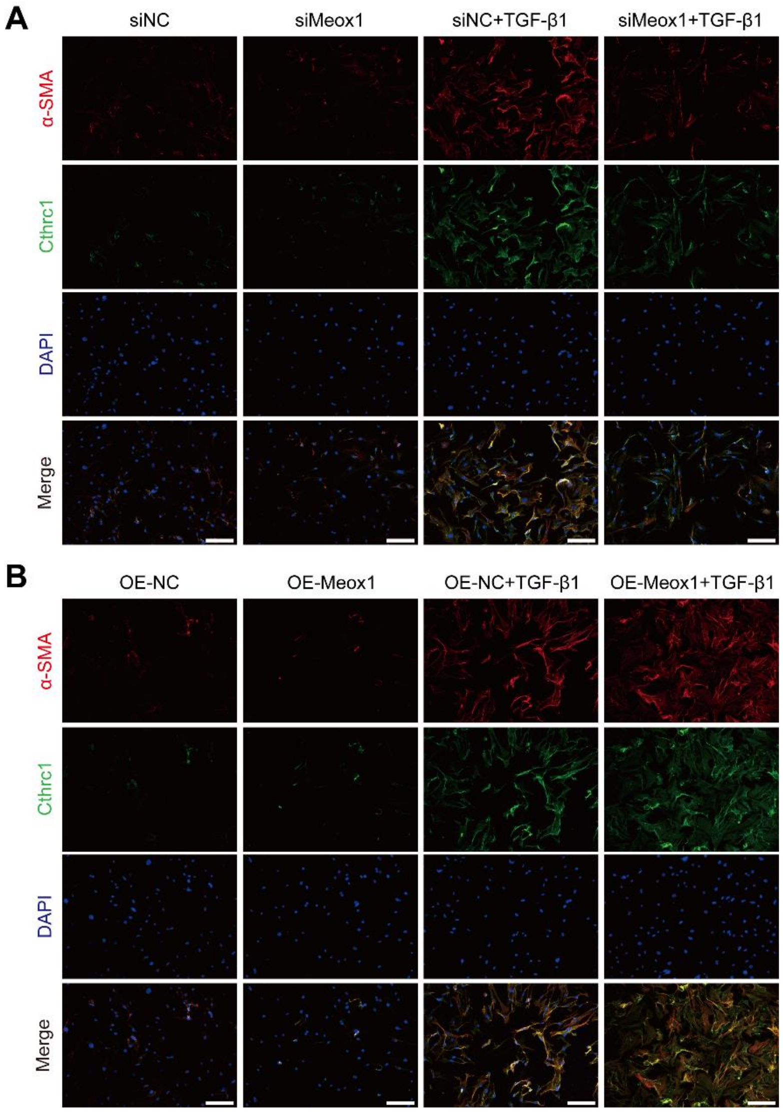

Figure S8. Meox1 regulated the expression of Cthrc1 in Myofbs. (A) Representative images of immunofluorescence staining of α-SMA (red) and Cthrc1 (green) in NMCFs transfected with siMeox1 or siNC followed by exposure to vehicle or TGF-β1. Scale bar, 100 μm. **(B)** Representative images of immunofluorescence staining of α-SMA (red) and Cthrc1 (green) in NMCFs transfected with OE-Meox1 or OE-NC followed by exposure to vehicle or TGF-β1. Scale bar, 100 μm.

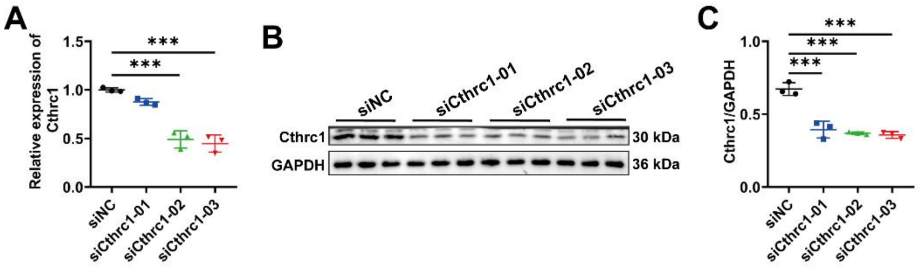

Figure S9. Knockdown efficacy of Cthrc1 siRNA in NMCFs. (A) RT-qPCR analysis of Cthrc1 mRNA level in NMCFs transfected with three independent siCthrc1 or siNC. n=3. **(B, C)** Representative immunoblot images and quantitative analysis of Cthrc1 protein level in NMCFs. n=3. Data are mean ± SD. ****P*<0.001 by one-way ANOVA followed by Dunnett post hoc test.

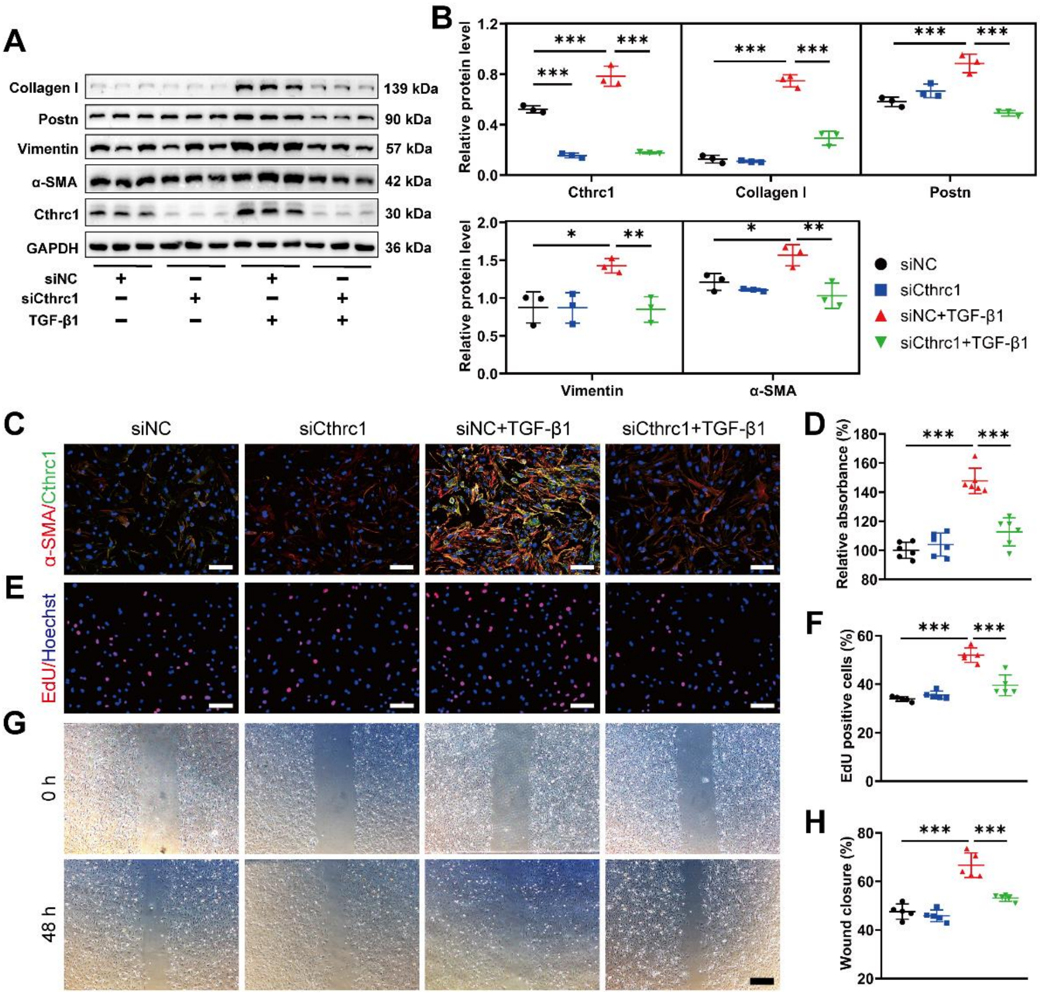

Figure S10. Silencing of Cthrc1 expression suppressed CFs-to-Myofbs phenotype

transformation upon TGF-β1 stimulation. (A, B) Representative immunoblot images and quantitative analyses of Cthrc1, Collagen I, Postn, Vimentin and α-SMA protein levels in NMCFs transfected with siCthrc1 or siNC followed by exposure to vehicle or TGF-β1. n=3. **(C)** Representative images of immunofluorescence staining of α-SMA (red) and Cthrc1 (green) in NMCFs. Scale bar, 100 μm. **(D)** Proliferation of NMCFs determined by CCK-8 assay. n=6. **(E, F)** EdU assay and quantitative analysis of the percentage of EdU-positive cells (red). Scale bar, 100 μm. n=5. **(G, H)** Representative micrographs of scratch wound of NMCFs and quantification of the area of scratch wound closure. n=5. Data are mean ± SD. **P*<0.05, ***P*<0.01, ****P*<0.001 by one-way ANOVA followed by Dunnett post hoc test.

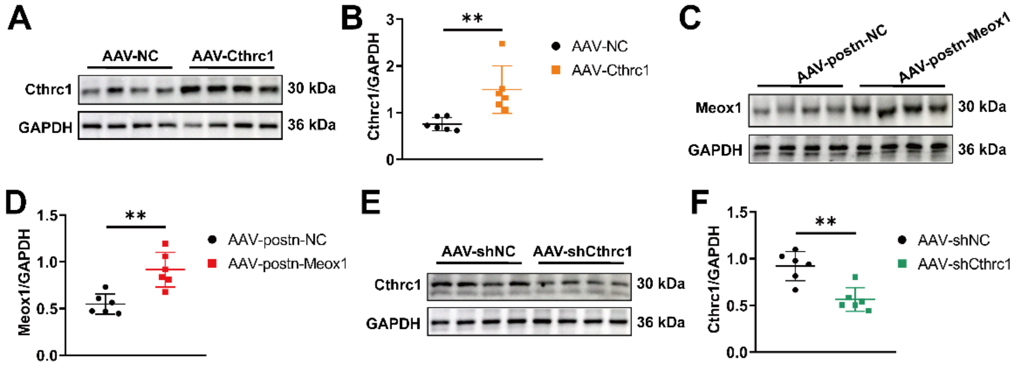

Figure S11. Validation of the transfection efficiency of AAV *in vivo*. (A, B) Representative immunoblot images and quantitative analysis of Cthrc1 protein level in mouse heart at day 28 after injection of the AAV vector expressing Cthrc1 or NC. n=6. **(C, D)** Representative immunoblot images and quantitative analysis of Meox1 protein level in fibroblasts isolated from hearts of MI mice injected with the AAV vector expressing Meox1 or NC. n=6. **(E, F)** Representative immunoblot images and quantitative analysis of Cthrc1 protein level in mouse heart at day 28 after injection of the AAV vector carried Cthrc1 shRNA. n=6. Data are mean ± SD. ***P*<0.01 by unpaired student's test.

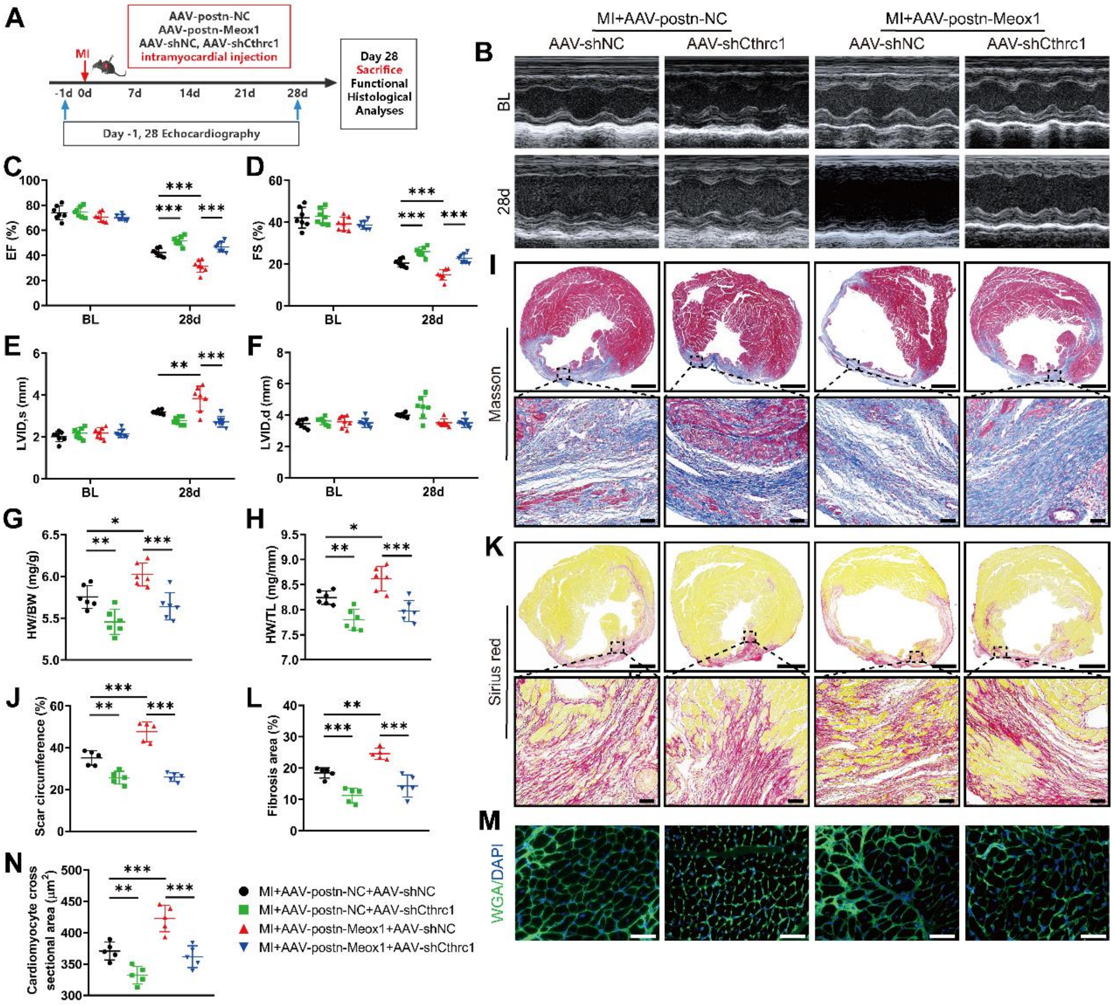

Figure S12. Silencing Cthrc1 blocked the aggravation of cardiac dysfunction and cardiac fibrosis induced by Meox1 overexpression in MI mice. (A) Schematic diagram of AAV-shCthrc1 or AAV-shNC and AAV-postn-Meox1 or AAV-postn-NC after MI. **(B)** Representative images of M-mode echocardiography at baseline and 28 days after MI. **(C-F)** LVEF, LVFS, LVID,s, and LVID,d. n=7. **(G, H)** The ratios of HW to BW and HW to TL. n=6. **(I, J)** Representative images and quantification of scar circumference of heart sections. Scale bars, 1000 μm (upper) and 100 μm (lower). n=5. **(K, L)** Representative images and quantification of fibrotic area of heart sections. Scale bars, 1000 μm (upper) and 50 μm (lower). n=5. **(M, N)** Representative images and quantification of cross-sectional area of cardiomyocyte of heart sections. Scale bar, 50 μm. n=5. Data are mean ± SD. *P<0.05, **P<0.01, ***P<0.001 by one-way ANOVA followed by Dunnett post hoc test.
